# Bacteria-based multiplex system eradicates recurrent infections with drug-resistant bacteria via photothermal killing and protective immunity elicitation

**DOI:** 10.1186/s40824-023-00363-0

**Published:** 2023-04-06

**Authors:** Youcui Xu, Yi Wu, Yi Hu, Mengran Xu, Yanyan Liu, Yuting Ding, Jing Chen, Xiaowan Huang, Longping Wen, Jiabin Li, Chen Zhu

**Affiliations:** 1 Medical Research Center, Guangdong Cardiovascular Institute, Guangdong Provincial People’s Hospital (Guangdong Academy of Medical Sciences), Southern Medical University, Guangzhou, 510080 Guangdong China; 2 Department of Radiology, The First Affiliated Hospital of USTC, Division of Life Sciences and Medicine, University of Science and Technology of China, Hefei, 230001 Anhui China; 3 Center for Biomedical Imaging, University of Science and Technology of China, Hefei, 230026 Anhui China; 4 Department of Oncology, The First Affiliated Hospital of USTC, Division of Life Sciences and Medicine, University of Science and Technology of China, Hefei, 230001 Anhui China; 5 Department of Infectious Diseases, The First Affiliated Hospital of Anhui Medical University, Hefei, 230022 Anhui China; 6 School of Life Sciences, Hefei Normal University, Hefei, 230601 Anhui China; 7 Department of Orthopaedics, Division of Life Sciences and Medicine, The First Affiliated Hospital of USTC, University of Science and Technology of China, Hefei, 230001 Anhui China

**Keywords:** Photosynthetic bacteria, Aluminum adjuvant, Rp@Al, Photothermal therapy, Protective antimicrobial immunity, Recurrent drug-resistant bacterial infections

## Abstract

**Background:**

The high mortality associated with drug-resistant bacterial infections is an intractable clinical problem resulting from the low susceptibility of these bacteria to antibiotics and the high incidence of recurrent infections.

**Methods:**

Herein, a photosynthetic bacteria-based multiplex system (Rp@Al) composed of natural *Rhodopseudomonas palustris* (Rp) and Food and Drug Administration-approved aluminum (Al) adjuvant, was developed to combat drug-resistant bacterial infections and prevent their recurrence. We examined its photothermal performance and in vitro and in vivo antibacterial ability; revealed its protective immunomodulatory effect; verified its preventative effect on recurrent infections; and demonstrated the system’s safety.

**Results:**

Rp@Al exhibits excellent photothermal properties with an effective elimination of methicillin-resistant *Staphylococcus aureus* (*MRSA*). In addition, Rp@Al enhances dendritic cell activation and further triggers a T helper 1 (T_H_1)/T_H_2 immune response, resulting in pathogen-specific immunological memory against recurrent *MRSA* infection. Upon second infection, Rp@Al-treated mice show significantly lower bacterial burden, faster abscess recovery, and higher survival under near-lethal infection doses than control mice.

**Conclusions:**

This innovative multiplex system, with superior photothermal and immunomodulatory effects, presents great potential for the treatment and prevention of drug-resistant bacterial infections.

**Graphical Abstract:**

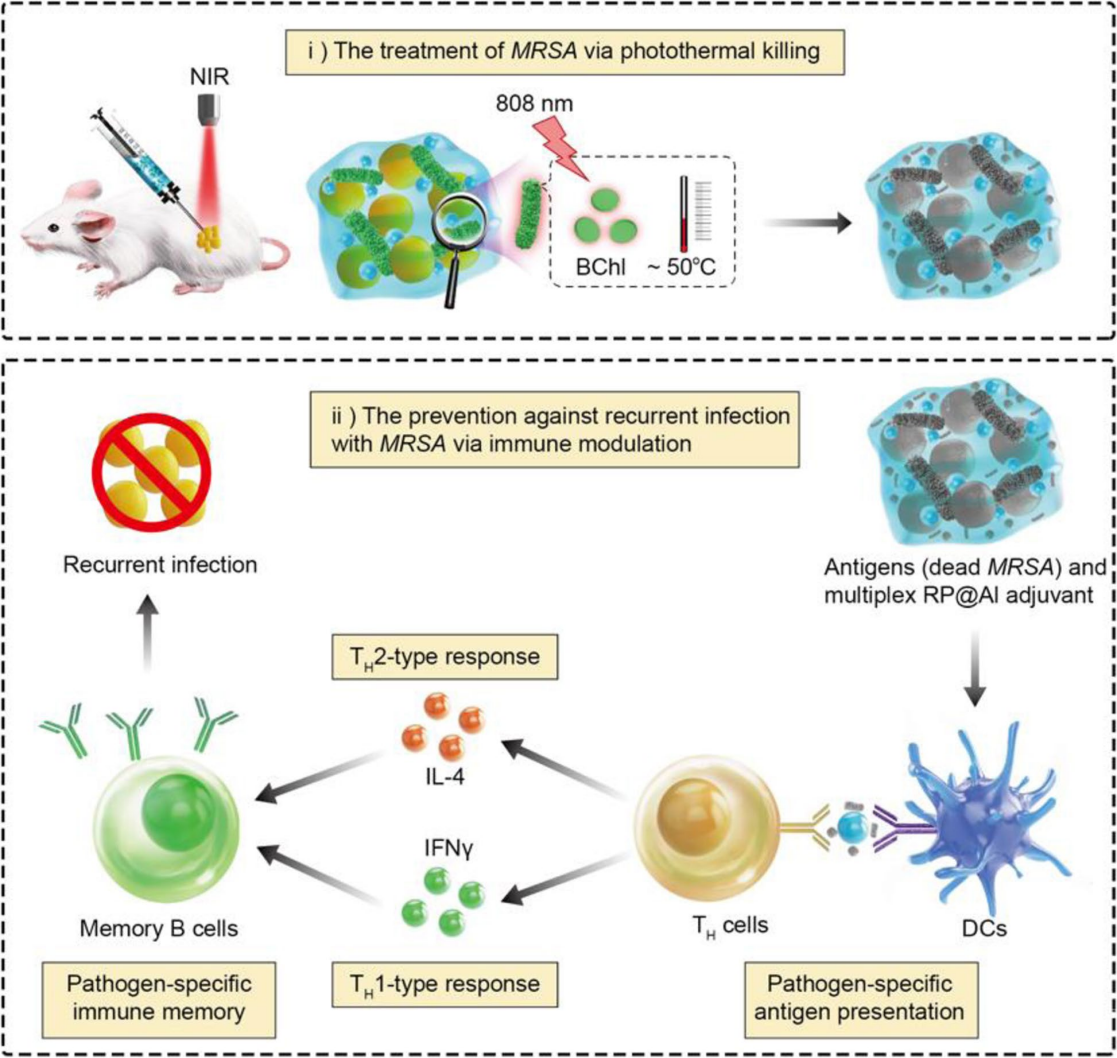

**Supplementary Information:**

The online version contains supplementary material available at 10.1186/s40824-023-00363-0.

## Background

In clinics, antibiotics are the preferred line of treatment for bacterial infections. However, their misuse has resulted in the emergence and rapid increase of multidrug-resistant pathogens [1–4]. A comprehensive study of 204 countries and territories estimated that there were 4.95 million deaths associated with antimicrobial resistance (AMR), which has become the third leading cause of death worldwide [5]. Furthermore, a review on AMR, commissioned by the UK government, reported that AMR-related deaths will exceed 10 million annually by 2050, surpassing cancer-related deaths [6]. The high mortality associated with drug-resistant bacterial infections is ascribed not only to the low susceptibility of these bacteria to antibiotics, but also to the high incidence of recurrent infections [7, 8]. Methicillin-resistant *Staphylococcus aureus* (*MRSA*), with a high risk of recurrence, is one of the most common drug-resistant bacteria in bacteremia, endocarditis, skin and soft tissue infections, bone and joint infections, and hospital-acquired infections [9]. Therefore, it is imperative to develop non-antibiotic therapies that integrate treatment and prevention against drug-resistant bacteria and their recurrent infections.

Bacteria-mediated therapies have attracted remarkable attention in recent years [10–15]. Bacteria possess considerable tumor-targeting ability because they can infiltrate tumors and surviving in the tumor hypoxic microenvironment, as well as immunoregulatory capacity owing to their immunogenicity [16–19]. Therefore, a series of bacteria-based strategies (including *Escherichia*, *Salmonella*, and *Listeria*) have been developed for cancer therapies [20–24]. In addition, probiotics (such as bifidobacteria and lactobacilli) with anti-inflammatory and immunomodulatory abilities are employed in the treatment of various gastrointestinal diseases, such as inflammatory bowel disease, acute pancreatitis, and *Helicobacter pylori* infections [25, 26]. Photosynthetic bacteria (PSB), the earliest photoautotrophic microorganisms, have been widely used as aquaculture feed because of their high nutrient content (including proteins, amino acids, vitamins, and bioactive substances) [27–29]. PSB, which have a primitive photoenergy synthesis system, carry out photosynthesis mainly through their bacteriochlorophyll (BChl), which converts sunlight into energy needed for metabolism. Importantly, BChl absorbs near-infrared (NIR) light with maximum absorption peaks around 805 and 865 nm, suggesting that PSB may be an innovative candidate for novel photothermal reagents [30–32]. Therefore, we believe that PSB-based photothermal therapy with less invasiveness and a lower risk of drug resistance has great potential against infections by drug-resistant bacteria, which has not been reported to date [33–36].

Host immunomodulatory therapies, which exploit the host-dependent natural mechanisms to activate or strengthen protective antimicrobial immunity, are a promising strategy for the prevention of drug-resistant bacterial infections [37–43]. A series of immunomodulators have been proposed for drug-resistant bacterial infections, including agonists that target natural immune pattern recognition receptors [PRRs; such as Toll-like receptors (TLRs) and NOD-like receptors (NLRs)] and immunomodulatory host defense peptides (also called antimicrobial peptides) [37, 44, 45]. Microbial signature components and endogenous damage-associated molecular patterns interact with PRRs, triggering the activation of mitogen-activated protein kinase and nuclear factor-κB signaling pathways, thereby activating innate immunity, and promoting the induction of antimicrobial effector functions [46, 47]. The activation of innate immunity is critical for the induction of adaptive immunity, which facilitates protective antimicrobial immunity. Agonists of TLRs and NLRs have been demonstrated to be safe and effective vaccine adjuvants, and the potential of immunomodulatory strategies against bacterial infections by targeting PRRs have also been explored [48, 49]. Food and Drug Administration-approved aluminum (Al) adjuvant, the most common NLRs agonist, has been reported to preferentially prime CD4^+^ T cells, which further activate B cells, thereby initiating an adaptive immunity response [50, 51]. Thus, immunomodulatory therapy is a potential approach to prevent recurrent infection with drug-resistant bacteria.

To achieve this, we integrated immunomodulatory therapy with bacteria-mediated photothermal therapy to achieve the following: i) develop a PSB-based multiplex system for treatment and prevention of drug-resistant bacterial infections; ii) demonstrate the photothermal killing effect of the system on drug-resistant bacteria; and iii) assess the immunomodulatory effect of the system in preventing recurrent infection with drug-resistant bacteria. We expect this study to provide guidelines for the design of bacteria-mediated antimicrobial systems and shed light on the development of “smart” systems that integrate bacterial treatment and prevention methodologies for clinical application in other AMR-related diseases.

## Methods

### Materials

Alhydrogel® adjuvant 2% was purchased from InvivoGen (San Diego, CA, USA). Brain Heart Infusion (BHI) Broth and BHI Agar were purchased from Qingdao Hope Bio-Technology Co., Ltd. (Qingdao, China). Crystal violet was purchased from Sangon Biotech Co., Ltd. (Shanghai, China). Dichloro-dihydro-fluorescein diacetate (DCFH-DA) was purchased from Beyotime Biotechnology (Shanghai, China). Masson’s trichrome staining kit was purchased from Beijing Solarbio Science & Technology Co., Ltd. (Beijing, China). Red blood cell lysis buffer and 4′,6-diamidino-2-phenylindole were purchased from Beyotime Biotechnology (Shanghai, China). All antibodies used in flow cytometry were purchased from BioLegend (San Diego, CA, USA). Interferon γ (IFNγ) and interleukin (IL)-4 enzyme-linked immunosorbent assay (ELISA) kits were purchased from Cusabio Biotechnology Co., Ltd. (Wuhan, China). Alanine aminotransferase (ALT), aspartate aminotransferase (AST), creatinine (CRE), blood urea nitrogen (BUN), total cholesterol (TC), triglyceride (TG), and glucose (GLU) assay kits were purchased from Nanjing Jiancheng Biological Engineering Institute (Nanjing, China).

### Cell culture

*Rhodopseudomonas palustris* (Rp) was purchased from the China Center of Industrial Culture Collection and cultured in Van Niel’s Yeast Agar medium in a liquid anaerobic environment at 30 °C with a 40 W tungsten-filament light for illumination. The *MRSA* reference strain (ATCC 43300) was obtained from the Anhui Center for Surveillance of Bacterial Resistance and cultured in BHI Broth in a table concentrator (220 rpm, 37 °C).

### Characterization of Rp

The morphology of Rp was characterized using a TH4-200 inverted optical microscope (Olympus, Tokyo, Japan) and a S-4800 scanning electron microscope (Hitachi, Ltd., Tokyo, Japan). The absorption spectrum of Rp was obtained using a UV–Vis spectrophotometer (Agilent Technologies, Santa Clara, CA, USA).

### Preparation and mechanism of Rp@Al

Rp was mixed with different concentrations (0.25%, 0.50%, 1.00%, 1.50%, and 2.00%) of Al adjuvant to form Rp@Al gel. Thereafter, the stability of Rp@Al was monitored at different time points (Days 0, 3, and 7). To examine the preparation of Rp@Al, we measured the zeta potentials of Rp, blocked Rp, and the Al adjuvant. We mixed Rp with positively charged polyethyleneimine to block the negative charge of Rp. The blocked Rp was then mixed with Al adjuvant to determine whether the Rp@Al system could be formed.

### Evaluation of the photothermal properties of Rp@Al

The photothermal properties of Rp@Al were evaluated by monitoring photothermal heating curves. Phosphate-buffered saline (PBS) and different concentrations of Rp (10^8^, 10^9^, and 10^10^ CFU/mL) were irradiated using an 808-nm laser with a power density of 2 W/cm^2^ for 8 min, and the photothermal heating curves were recorded. Photothermal heating curves were also recorded for Rp (10^9^ CFU/mL) exposed to various laser power densities (0.5, 1.0, 1.5, and 2.0 W/cm^2^). The photothermal stability of Rp@Al was further investigated by repeated off-and-on laser irradiation (2 W/cm^2^ for 8 min, cooling for 10 min, four cycles).

### Evaluation of antibacterial activity in vitro

Five experimental groups were defined as follows: control, Rp + NIR, Al, Rp@Al, and Rp@Al + NIR. *MRSA* (10^7^ CFU/mL or 10^10^ CFU/mL) was cultured in the presence of PBS, Rp (10^9^ CFU/mL), Al adjuvant (2%), and Rp@Al (gel formed by 10^9^ CFU/mL Rp and 2% Al adjuvant), respectively. The Rp + NIR and Rp@Al + NIR groups were exposed to an 808-nm laser with a power density of 2 W/cm^2^ for 8 min. For colony counting analysis, the bacteria in each group were cultured for an additional 1 h at 37 °C after different treatments; thereafter, dilutions of the cultures were plated on BHI Agar and cultured at 37 °C for 24 h. Then, the colonies were counted and analyzed using the ImageJ software. For anti-biofilm analysis, after different treatments, the bacteria in each group were cultured for an additional 12 h at 37 °C to allow biofilm growth; thereafter, crystal violet staining was conducted according to the manufacturer’s instructions. In addition, to determine the reactive oxygen species (ROS) levels in the *MRSA* (10^10^ CFU/mL) after the various treatments, DCFH-DA staining was performed according to the manufacturer’s protocol. After staining, diluents of the mixture in each treatment group were coated on slides for fluorescence microscope imaging.

### Animals

BALB/c mice (6–8 weeks old) were purchased from Nanjing Pharmaceutical Factory Co. Ltd. (Nanjing, China).

### Evaluation of antibacterial activity and immunity response in vivo

BALB/c mice were subcutaneously (s.c.) injected with *MRSA* (10^9^ CFU) to establish a cutaneous abscess model. The mice were then divided into five groups (*n* = 5 per group): control, Rp + NIR, Al, Rp@Al, and Rp@Al + NIR. On Day 2, mice with established abscesses were injected in situ with PBS, Rp (10^9^ CFU), Al adjuvant (2%), or Rp@Al (the gel comprising 10^9^ CFU Rp and 2% Al adjuvant) at the abscess site. Mice in the Rp + NIR and Rp@Al + NIR groups were exposed to an 808-nm laser with a power density of 1.5 W/cm^2^ for 8 min. After different treatments, abscess recovery and body weight were monitored every two days, and mice were euthanized on Day 12.

To evaluate antibacterial activity, we collected residual bacteria from the abscess sites by homogenization in a tissue homogenizer (Bertin, Montigny le Bretonneux, France). Dilutions of the supernatants were plated on BHI Agar and cultured at 37 °C for 24 h; then, the colonies were counted and analyzed with ImageJ software. To evaluate the wound healing process, skin samples collected at the abscess sites were fixed in 4% paraformaldehyde (PFA) for Masson’s trichrome staining according to the manufacturer’s instructions. Serum and the sentinel lymph nodes were collected to assess the immune response. Serum IFNγ and IL-4 levels were determined by ELISA according to the manufacturer’s instructions. Single-cell suspensions of lymph nodes were blocked with CD16/32 antibody and then divided into two parts, labeled with the following antibodies separately: FITC-CD45.2 (104), PE/Cy7-CD11c (N418), APC-CD80 (16-10A1), and PE-CD86 (A17199A); FITC-CD45.2 (104), and APC-CD4 (GK1.5). The activation of dendritic cells (DCs) and the percentage change of CD4^+^ T cells in lymph nodes were analyzed using a CytoFLEX flow cytometer (Beckman Coulter, Brea, CA, USA).

### Evaluation of protective antimicrobial immunity induction and recurrent infection prevention

To evaluate the induction of protective antimicrobial immunity, BALB/c mice with established abscesses were treated as described above and euthanized after 4 weeks. Serum, sentinel lymph nodes, and spleens were collected to assess the immune response. Serum IFNγ and IL-4 levels were determined by ELISA according to the manufacturer’s instructions. Single-cell suspensions of lymph nodes and spleens were blocked with CD16/32 antibody and then labeled with APC/Cyanine7-CD45.2 (104) and PE-CD45R/B220 (RA3-6B2). The percentage change of B220^+^ B cells in lymph nodes and spleens was analyzed using the CytoFLEX flow cytometer.

To evaluate the prevention of recurrent infections, BALB/c mice with established abscesses were reared normally for 4 weeks after the initial treatment, as described above. Specifically, PBS, Rp (10^9^ CFU), Al adjuvant (2%), or Rp@Al (a gel comprising 10^9^ CFU Rp and 2% Al adjuvant) were injected in situ at the abscess site on day 2. Mice in the Rp + NIR and Rp@Al + NIR groups were subsequently exposed to an 808-nm laser with a power density of 1.5 W/cm^2^ for 8 min. After 4 weeks, mice in each group were then given a second s.c. administration of *MRSA* (1 × 10^9^ CFU) or intravenous (i.v.) administration of *MRSA* (7.5 × 10^8^ CFU). For the s.c. recurrent infection model, abscess recovery was monitored every 3 days, and all mice were euthanized after 12 days. Colony counting at the abscess site was performed to evaluate the antibacterial activity, and skin samples from abscess sites were fixed in 4% PFA for Masson’s trichrome staining to evaluate the wound healing process. For the i.v. recurrent infection model, the body weight of mice was monitored every day for 3 weeks, and the survival period of mice in each group was analyzed.

### In vivo safety assessment

After treatment, BALB/c mice in each group were euthanized on Day 12, and the serum, heart, liver, spleen, lungs, and kidneys were collected for biochemical indicator assessment and histological evaluation. Serum biochemical indicators, including ALT, AST, CRE, BUN, TC, TG, and GLU levels, were measured using assay kits according to the corresponding manufacturer’s protocol. In addition, for histological evaluation, hematoxylin and eosin (H&E) staining of tissue sections was performed, and the tissue structure was further analyzed.

### Statistical analysis

Data are shown as mean ± SD or mean ± SEM; *n* = 5–10. Statistical differences were analyzed using Student’s *t*-test (two-tailed) and indicated as ns, no significance, * P < 0.05, ** P < 0.01, *** P < 0.001, and ****P < 0.0001.

## Results

### Preparation and characterization of Rp@Al

**Fig. 1:**
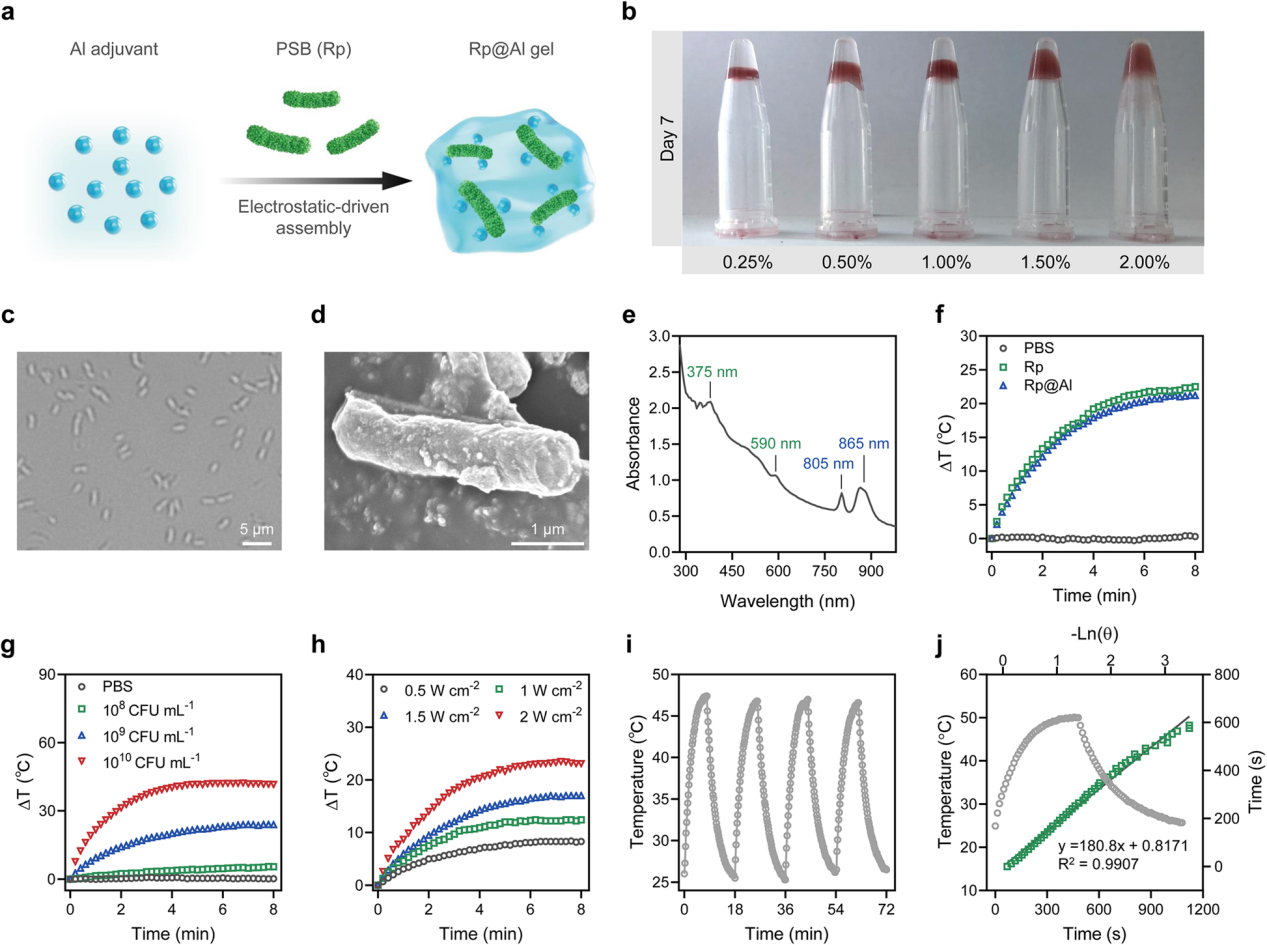
Preparation and characterization of Rp@Al with photothermal performance. **a** Schematic diagram of the preparation of Rp@Al composed of *Rhodopseudomonas palustris* (Rp) and aluminum (Al) adjuvant. **b** Stability of Rp@Al gel on Day 7. **c**, **d** Representative optical microscope (**c**) and scanning electron microscope (**d**) images of Rp. **e** Absorption spectrum of live Rp. **f** Photothermal temperature curves of phosphate-buffered saline (PBS), Rp, and Rp@Al under an 808-nm laser with a power density of 2 W/cm^2^ for 8 min. **g** Photothermal temperature curves of PBS and different concentrations of Rp@Al under an 808-nm laser with a power density of 2 W/cm^2^ for 8 min. **h** Photothermal temperature curves of Rp@Al under an 808-nm laser with different power densities for 8 min. **i** Photothermal stability of Rp@Al under an 808-nm laser with a power density of 2 W/cm^2^ for four irradiation/cooling cycles. **j **Photothermal conversion efficiency of Rp@Al

The PSB used in this study was Rp, a gram-negative bacterium. Interestingly, Rp could interact with different concentrations of Al adjuvant to form Rp@Al gel in a few seconds (Fig. 1a; Fig. S1), which may be ascribed to colloid homeostasis due to charge adsorption [52]. Validating this deduction, the average zeta potentials of Rp and the Al adjuvant were found to be − 9.44 and + 11.30 mV (Fig. S2a), respectively. Positively charged polyethyleneimine was mixed with Rp to block its negative charge. Blocked Rp, with an average zeta potential of + 2.06 mV (Fig. S2a), was then mixed with Al adjuvant. Interestingly, Rp shielded from a negative charge was unable to form a composite gel system with the Al adjuvant (Fig. S2b). Different concentrations of Al adjuvant mixed with Rp formed gels with different viscosities, among which the gel formed by 2% Al adjuvant and Rp displayed good stability even for 7 days (Fig. 1b; Fig. S1). Optical microscope and scanning electron microscope images showed that Rp has a uniform cylindrical shape, with an average length of 2.6 μm (Fig. 1c, d; Fig. S3). Importantly, BChl contained in Rp showed NIR absorption at 700–1,000 nm, which is different from the visible light absorption window of plant chlorophyll. The live Rp culture appeared dark red under normal growth conditions (Fig. S4), exhibiting characteristic absorption peaks of BChl at around 805 and 865 nm, and absorption peaks of carotenoid at approximately 375 and 590 nm (Fig. 1e), which was consistent with a previous report [32]. The photothermal properties of Rp@Al were investigated under an 808-nm laser irradiation. As expected, Rp@Al exhibited excellent photothermal performance similar to that of Rp (Fig. 1f; Fig. S5). Moreover, Rp@Al showed concentration-dependent and power density-dependent photothermal effects, as indicated by photothermal images and heating curves (Fig. 1g, h; Figs. S6 and S7). In addition, Rp@Al presented commendable photothermal stability under repeated off-and-on laser irradiation (Fig. 1i), where the photothermal conversion efficiency of Rp@Al was calculated to be 36.20%, according to the method reported, which is higher than that of various photothermal agents reported to date (Fig. 1j; Table S1) [32, 53–56]. Thus, Rp@Al with prominent photothermal properties and stability was successfully prepared.

### Photothermal antibacterial activity of Rp@Al in vitro

**Fig. 2:**
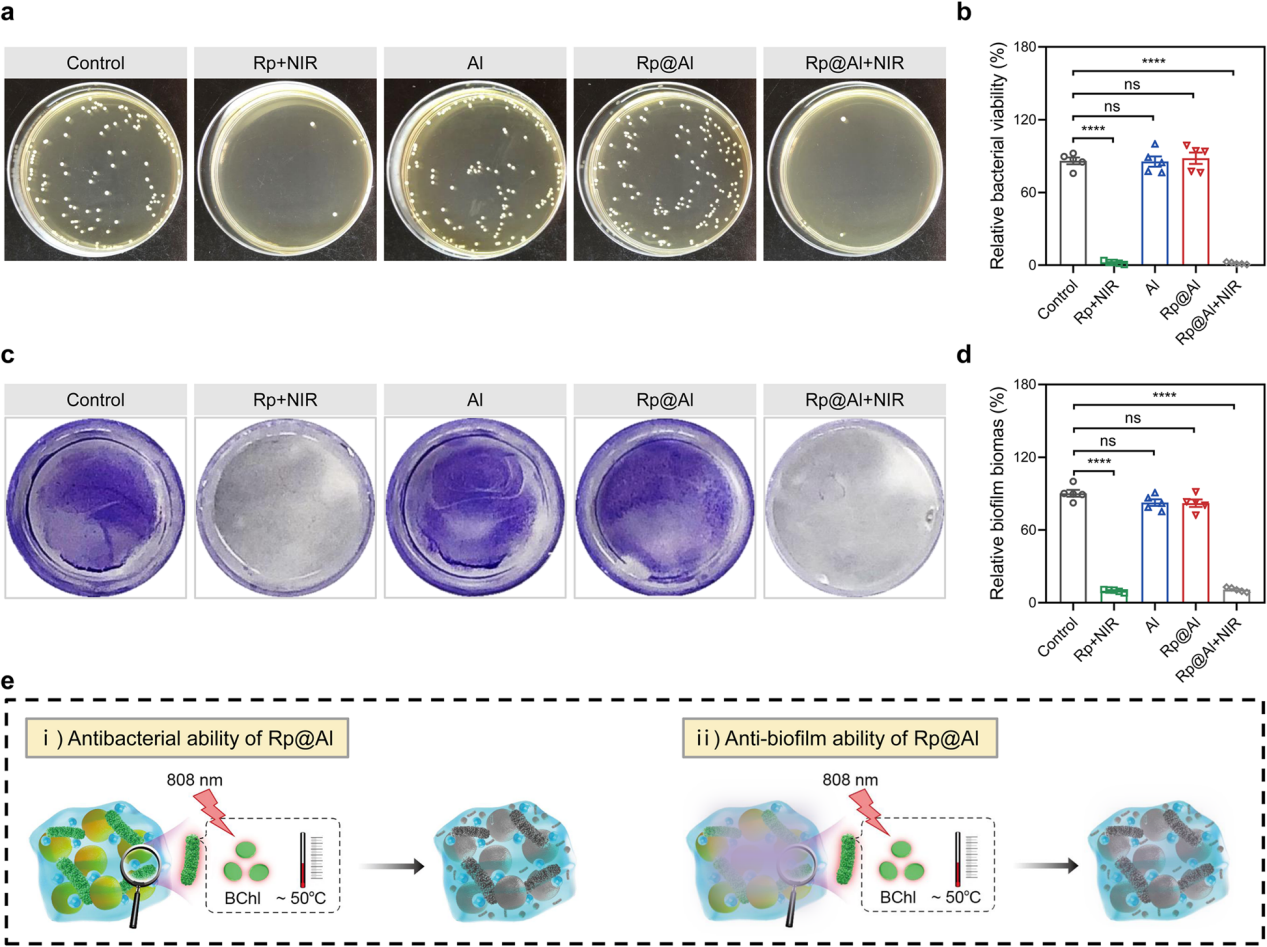
Antibacterial activity of Rp@Al in vitro. **a**, **b** Culture plate images (**a**) and quantitative analysis (**b**) showing the antibacterial ability of different treatments (mean ± SD, *n* = 5). **c**, **d** Crystal violet staining images (**c**) and quantitative analysis (**d**) showing the anti-biofilm ability of different treatments (mean ± SD, *n* = 5). **e** Schematic diagram showing the antibacterial and anti-biofilm performance of Rp@Al. ns, no significance, ****P < 0.0001

Encouraged by the photothermal performance of Rp@Al, we next examined the antibacterial and anti-biofilm efficiencies of Rp@Al using gram-positive *MRSA*. After irradiation at 2 W/cm^2^ for 8 min, the relative bacterial viability in the Rp + NIR and Rp@Al + NIR groups was only 2.11% and 1.56%, respectively. The antibacterial efficiency of Al and Rp@Al alone was also examined, and the results showed that Al and Rp@Al alone had no obvious antibacterial effect (Fig. 2a, b), indicating that the antibacterial activity of Rp@Al should be attributed to its photothermal properties. Bacterial biofilm is one of the causes of antibiotic tolerance. In this regard, the *MRSA* biofilm disruption after different treatments was verified using crystal violet staining. Compared with those in the control group, biofilms were severely damaged in the Rp + NIR and Rp@Al + NIR groups, while there was no significant difference in Al and Rp@Al groups (Fig. 2c, d). These findings suggested that Rp@Al exhibited a potent anti-biofilm efficiency against *MRSA* through its photothermal effect. Local hyperthermia may destroy bacterial structures by producing ROS or denaturing protein structure, ultimately resulting in bacterial death [57]. Hence, DCFH-DA staining was performed to reveal the ROS levels in the *MRSA* after the various treatments (Fig. S8). The results suggest that the photothermal antibacterial mechanism of Rp@Al may be attributed to the production of ROS that destroy bacterial structures, eventually killing the bacteria. In conclusion, the Rp@Al developed in this study integrates the advantages of organic and inorganic nanomaterials for antibacterial applications, as summarized in Table S2 [58, 59], exhibiting prominent photothermal antibacterial and anti-biofilm abilities (Fig. 2e).

### Rp@Al exerts excellent antibacterial activity and enhances the T_H_-type immune response in vivo

**Fig. 3:**
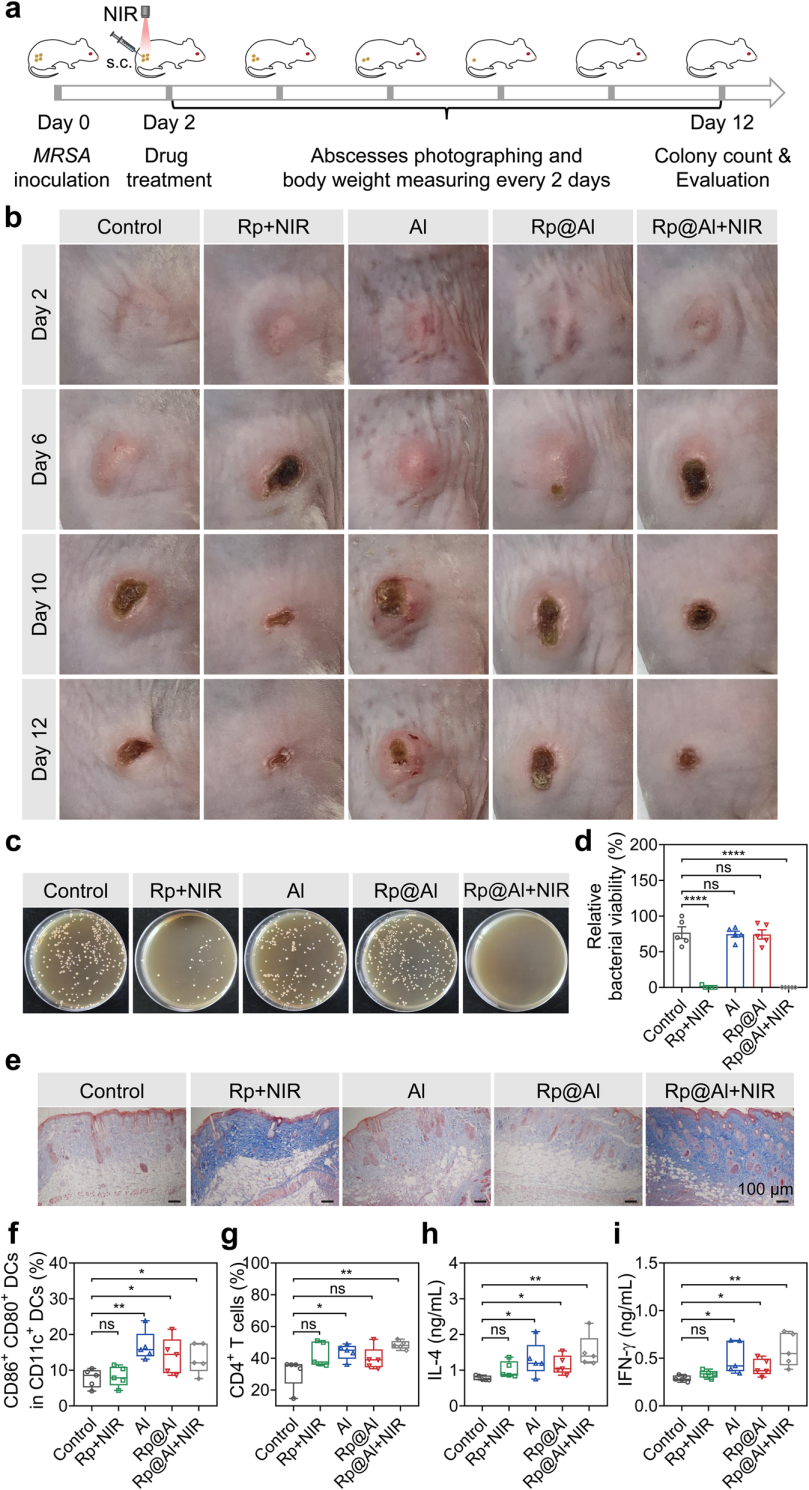
Evaluation of Rp@Al-based antibacterial activity and immune response in vivo. **a** Schematic illustration of abscess model establishment and therapeutic strategy. **b **Time-lapse images of methicillin-resistant *Staphylococcus aureus* (*MRSA*)-induced abscess recovery after different treatments. **c**, **d **Culture plate images (**c**) and quantitative analysis (**d**) showing the in vivo antibacterial ability of different treatments (mean ± SEM, *n* = 5). **e** Masson’s trichrome staining of skin tissues at abscess sites after different treatments. **f** Percentage of CD86^+^ CD80^+^ dendritic cells (DCs) in CD11c^+^ DCs in lymph nodes from mice in different treatment groups (mean ± SEM, *n* = 5). **g** Percentage of CD4^+^ T cells in total leukocytes isolated from lymph nodes harvested from different treatment groups (mean ± SEM, *n* = 5). **h**, **i** Quantitative analysis of serum interleukin (IL)-4 (**h**) and interferon γ (IFNγ) (**i**) levels on Day 12 (mean ± SEM, *n* = 5). ns, no significance, *P < 0.05, **P < 0.01, and ****P < 0.0001

We next explored the antibacterial activity of Rp@Al and the immune response elicited by Rp@Al in mice with cutaneous abscesses generated by s.c. administration of *MRSA*. On Day 2, the different treatments were administered; the local temperature of the abscess site rose to approximately 49 °C in the Rp + NIR and Rp@Al + NIR groups (Fig. S9). The abscesses were photographed and body weights were measured every 2 days. All mice were euthanized at the end of treatment (Day 12), and the antibacterial activity and immune response were evaluated (Fig. 3a). Images of abscesses showed that the recovery in the Rp + NIR and Rp@Al + NIR groups was better than that in the control group, while the abscesses in the Al and Rp@Al groups showed no significant recovery (Fig. 3b), corroborating the commendable photothermal antibacterial ability of Rp@Al. To validate this, we performed bacterial colony counting at the abscess site. As expected, the results showed that compared with that in the control group, the relative bacterial viability in the Rp + NIR and Rp@Al + NIR groups was significantly decreased and was only 0.85% and 0.13%, respectively, whereas that in the Al and Rp@Al groups showed no significant difference (Fig. 3c, d). In addition, Masson’s trichrome staining of the skin at the abscess site showed more collagen deposition in the Rp + NIR and Rp@Al + NIR groups than that in the control group, while the collagen deposition in the Al and Rp@Al groups showed no significant change (Fig. 3e; Fig. S10). This confirmed that Rp@Al effectively promotes abscess recovery via its distinguished photothermal antibacterial ability.

To assess the immune response elicited by Rp@Al, we collected sentinel lymph nodes and serum from the euthanized mice for analysis. We first evaluated the activation of DCs, the main antigen-presenting cells. Notably, compared with that in the control group, the percentage of CD86^+^ CD80^+^ DCs in CD11c^+^ DCs was significantly increased in both the Al, Rp@Al, and Rp@Al + NIR groups, while that in the Rp + NIR group showed no significant increase (Fig. 3f). The proportion of CD4^+^ T cells in the Al, Rp@Al, and Rp@Al + NIR groups was also upregulated compared with that in the control group (Fig. 3g; Fig. S11). Together, these findings indicated that Rp@Al may have enhanced the antigen presentation of activated DCs to prime naive CD4^+^ T cells. Polarization of CD4^+^ T cells into T helper 1 (T_H_1) and T_H_2 is crucial for induction of protective antimicrobial immunity [60]. In view of this, the levels of IL-4 and IFNγ in serum were determined using ELISA. IL-4 and IFNγ production are critical features of T_H_2 and T_H_1 cell responses, respectively [60]. The levels of IL-4 and IFNγ in the Al, Rp@Al, and Rp@Al + NIR groups increased significantly, while neither the IL-4 nor IFNγ level in the Rp + NIR group showed significant differences when compared with those in the control group (Fig. 3h, i). This initiation of T_H_-type immune response induced by Rp@Al may be attributed to the Al adjuvant, as they are reported to prime T_H_ cell responses preferentially [50]. In conclusion, Rp@Al elicited not only prominent antibacterial activities but also enhanced T_H_1/T_H_2 immune response.

### Adjuvant-like Rp@Al effectively prevents recurrent infection by inducing pathogen-specific immunological memory

**Fig. 4:**
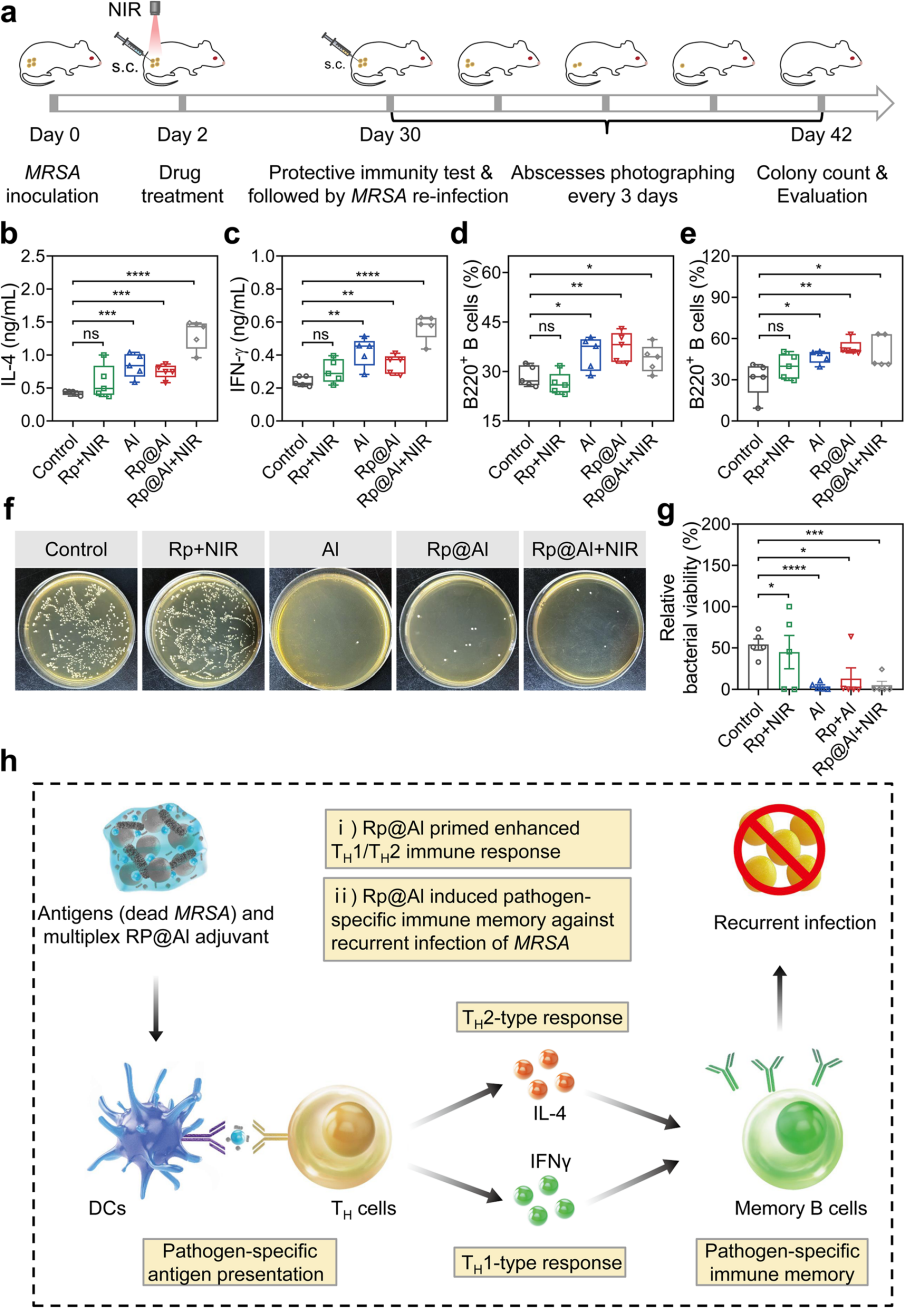
Rp@Al-based protective immunity prevents *MRSA*-induced recurrent infections by eliciting pathogen-specific immunological memory. **a** Schematic illustration of the evaluation of protective immunity and prevention of *MRSA*-induced recurrent infections. Mice were injected subcutaneously (s.c.) with *MRSA* (10^9^ CFU, first exposure) followed by different treatments on Day 2. After 4 weeks, serum, lymph nodes, and spleens of mice in different treatment groups were collected for protective immunity analysis. Prevention of recurrent infections was evaluated by s.c. re-infection with *MRSA*(10^9^ CFU, second exposure). **b**, **c** Quantitative analysis of serum IL-4 (**b**) and IFNγ (**c**) levels on Day 30 (mean ± SEM, *n* = 5). **d**, **e** Percentage of CD45^+^ B220^+^ memory B cells in lymph nodes (**d**) and spleens (**e**) from mice in different treatment groups on Day 30 (mean ± SEM, *n* = 5). **f**, **g** Culture plate images (**f**) and quantitative analysis (**g**) showing the antibacterial ability of different treatments after s.c. re-infection with *MRSA* (mean ± SEM, *n* = 5). **h** Schematic diagram showing the mechanism of Rp@Al-based prevention of recurrent infections with *MRSA*. ns, no significance, *P < 0.05, **P < 0.01, ***P < 0.001, and ****P < 0.0001

Given that Rp@Al induced T_H_-type immune responses, we next evaluated the generation of immune memory in abscess-bearing mice after Rp@Al treatment. BALB/c mice with *MRSA*-infected abscesses were grouped and treated as described above (Fig. 4a). We first verified the T_H_1/T_H_2 immune responses primed by Rp@Al by measuring serum levels of IL-4 and IFNγ using ELISA. Compared with those in the control group, the levels of IL-4 and IFNγ in the Al, Rp@Al, and Rp@Al + NIR groups were significantly increased, while they were not significantly different in the Rp + NIR group (Fig. 4b, c). Furthermore, memory B cells in sentinel lymph nodes and spleens were analyzed by flow cytometry. Compared with those in the control group, the percentages of B220^+^ B cells in lymph nodes and spleens in the Al, Rp@Al, and Rp@Al + NIR groups were significantly increased, while those in the Rp + NIR group showed no significant difference (Fig. 4d, e; Fig. S12), suggesting that immune memory was evoked after Rp@Al treatment.

To evaluate the preventive effect of immune memory against recurrent infections after initial Rp@Al treatment, we reared mice normally for 4 weeks and then administered a second dose of *MRSA* s.c. and monitored abscess recovery as described (Fig. 4a). Interestingly, bacterial counts were lower and abscess recovery was faster in the Al, Rp@Al, and Rp@Al + NIR groups than in the Rp + NIR and control groups (Fig. 4f, g; Fig. S13). Additionally, Masson’s trichrome staining of the skin at the abscess site showed more collagen deposition in the Al, Rp@Al, and Rp@Al + NIR groups than in the Rp + NIR and control groups (Fig. S14), further demonstrating the enhanced abscess healing after Rp@Al treatment. Together, these findings suggested that a protective antimicrobial immunity evoked by Rp@Al could effectively prevent recurrent infections with drug-resistant bacteria (Fig. 4h).

**Fig. 5:**
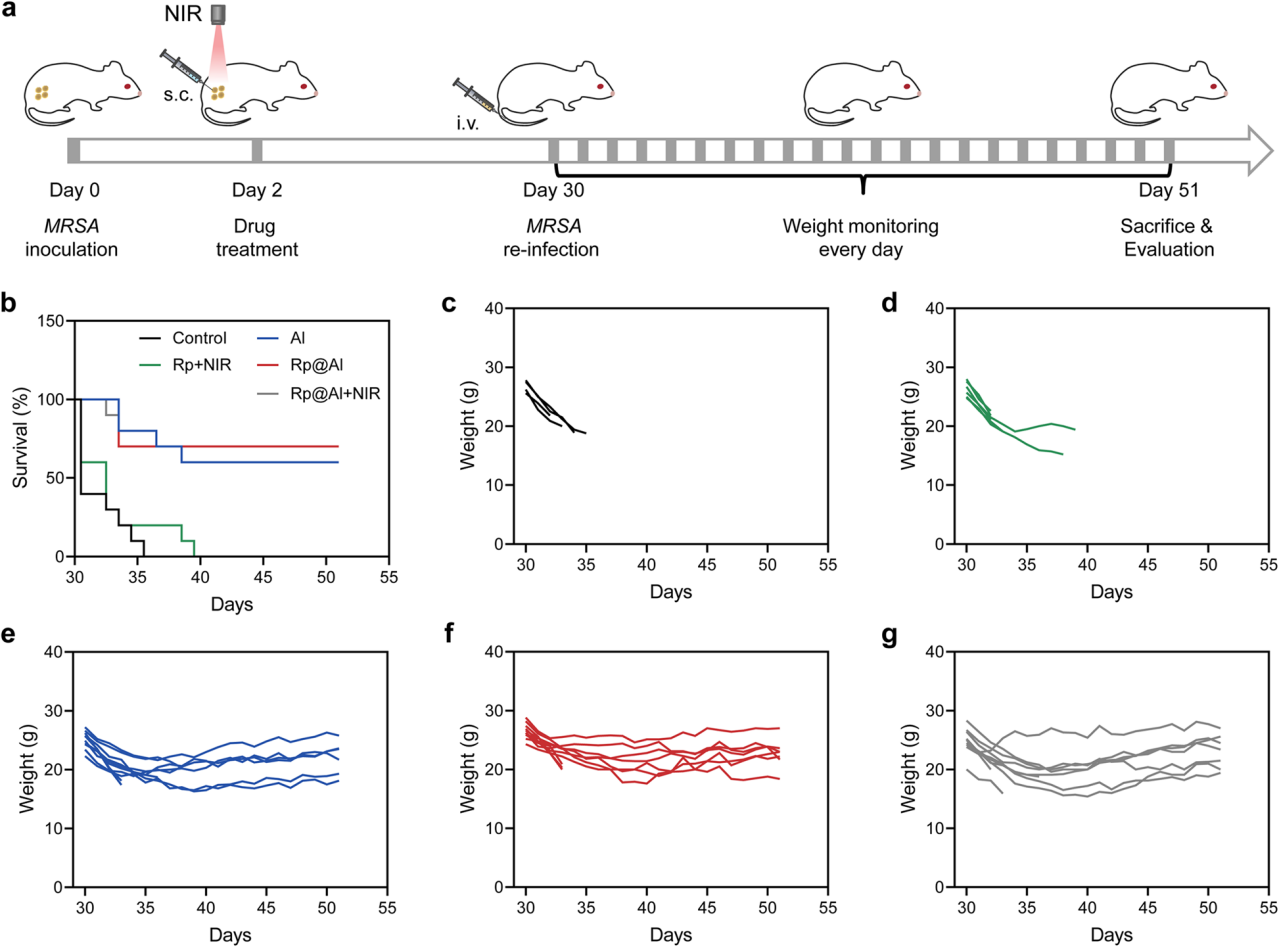
Rp@Al-based prevention of *MRSA*-induced recurrent infections and improvement of the survival rate. **a** Schematic representation illustrating the prevention of *MRSA*-induced recurrent infections. Mice were injected s.c. with *MRSA* (10^9^ CFU, first exposure) followed by different treatments on Day 2. After 4 weeks, prevention of recurrent infections was evaluated by i.v. re-infection with *MRSA* (7.5 × 10^8^ CFU, second exposure). **b **Survival curve of mice in different treatment groups after i.v. re-infection with *MRSA* (*n* = 10). **c**–**g **Body weight monitoring of mice in different treatment groups after i.v. re-infection with *MRSA* (*n* = 10)

To further confirm the protective effect of pathogen-specific immunological memory elicited by Rp@Al, mice were housed for 4 weeks after the initial treatment and were then i.v. re-infected with *MRSA* at a near-lethal infection dose. Body weight and the survival of mice in each group were monitored (Fig. 5a). No significant difference was observed in the initial body weight of mice among the different groups (Fig. S15). Encouragingly, compared with that in the control and Rp + NIR groups, the survival rate of mice in the Al, Rp@Al, and Rp@Al + NIR groups was significantly increased by up to 60–70%, and the survival period of mice in these groups was also significantly increased (Fig. 5b). Specifically, mice in both the control and Rp + NIR groups rapidly lost weight and did not survive past 5 and 9 days, respectively (Fig. 5c, d), whereas the weight of mice in the Al, Rp@Al, and Rp@Al + NIR groups returned to the normal range after approximately 1 week (Fig. 5e–g), although the body weight of mice in each group showed a downward trend upon second infection. In conclusion, adjuvant-like Rp@Al effectively prevented recurrent infections with drug-resistant bacteria by inducing pathogen-specific immunological memory.

### Biosafety assessment of Rp@Al

**Fig. 6:**
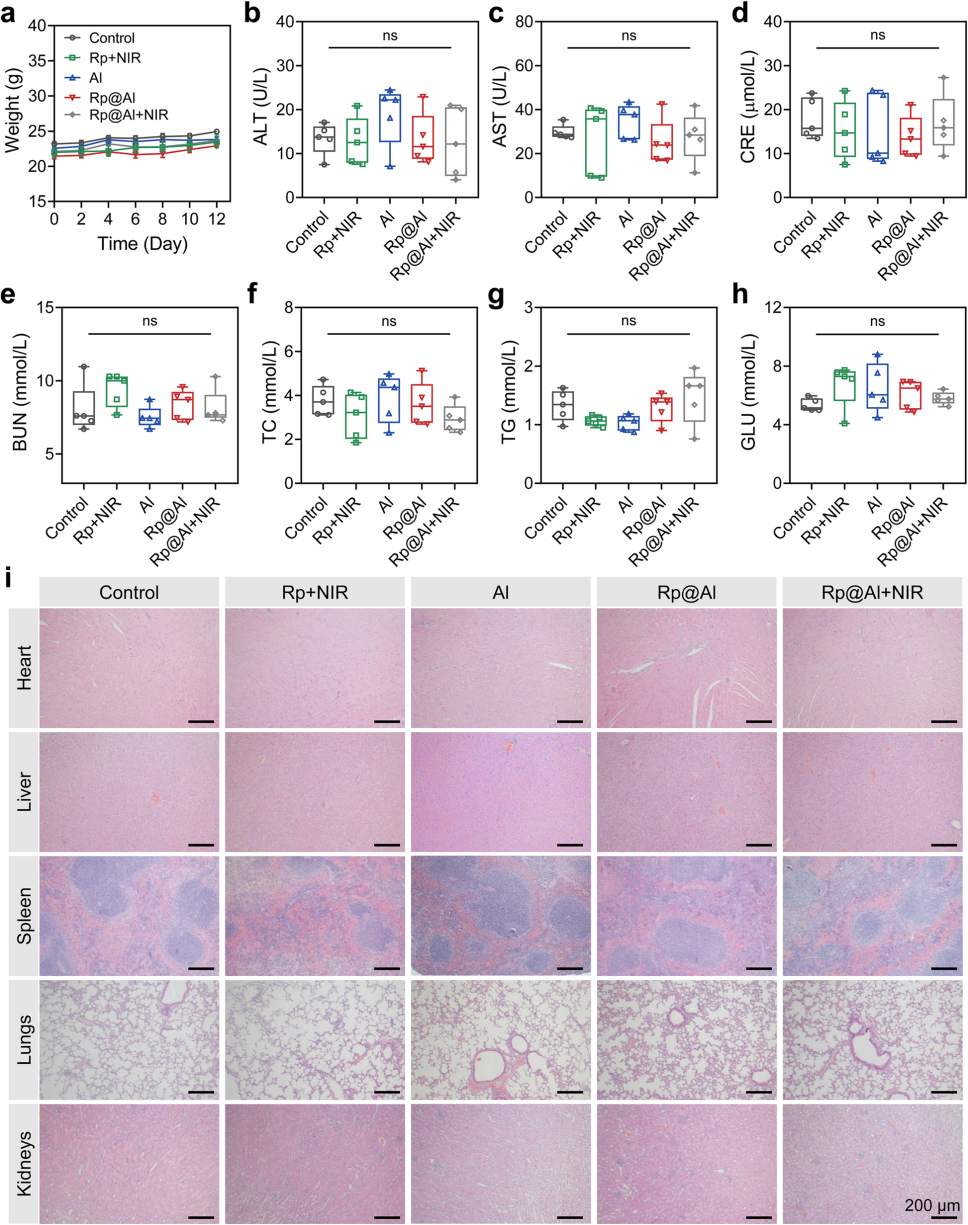
In vivo biosafety evaluation of Rp@Al. **a **Body weight monitoring of mice in different treatment groups. **b**–**h **Analysis of serum biochemical indicators of mice from different groups at the end of treatment (mean ± SEM, *n* = 5). **i** Representative hematoxylin and eosin (H&E) staining images showing the major organ tissue structures of mice in different treatment groups. ns, no significance

We next evaluated the biosafety of the multiplex system. The weight of mice in each treatment group did not show a downward trend, indicating the biosafety of the treatment system to some extent (Fig. 6a). Mice were euthanized at the end of treatment for the analysis of biochemical indicators and organ safety. There were no significant differences in serum ALT and AST levels among the Rp + NIR, Al, Rp@Al, and Rp@Al + NIR groups compared with those in the control group (Fig. 6b, c), indicating that the treatment system did not affect the liver function of mice. Moreover, there were no significant differences in CRE and BUN levels among the Rp + NIR, Al, Rp@Al, and Rp@Al + NIR groups when compared with those in the control group (Fig. 6d, e), suggesting that the treatment system had no significant influence on the renal function of mice. TC, TG, and GLU levels in mice in each treatment group also showed no significant differences, further confirming the biosafety of the treatment system (Fig. 6f–h). Furthermore, the evaluation of organ safety of the treatment system revealed no systematic toxicity to the heart, liver, spleen, lungs, and kidneys of mice in each treatment group, as assessed by H&E staining (Fig. 6i). Taken together, these findings suggested the biosafety of the multiplex system prepared herein.

## Discussion

The high mortality associated with drug-resistant bacteria and its recurrent infections is an intractable clinical problem [61]. It is challenging to achieve the treatment of drug-resistant bacteria and the prevention of their recurrent infection simultaneously using existing strategies [37, 44, 45, 62]. In this study, a PSB-based multiplex system, Rp@Al, was successfully developed for the treatment and prevention of drug-resistant bacterial infections. Rp@Al, containing BChl that absorbs NIR light at around 805 and 865 nm, exhibited prominent stability and photothermal capability, with a photothermal conversion efficiency of 36.20% and up to 98.44% antibacterial efficacy in vitro*.* Furthermore, in a *MRSA*-infected cutaneous abscess model, we demonstrated that Rp@Al also displayed a predominant photothermal antibacterial effect, effectively enhancing abscess recovery. Taken together, these results suggested that the PSB-based Rp@Al can be used as a novel photothermal reagent, which has great potential for photothermal treatment of drug-resistant bacterial infections.

Immunomodulatory therapy, which exploit host-dependent natural mechanisms to activate or strengthen protective antimicrobial immunity, is a potential approach for preventing recurrent infection with drug-resistant bacteria [37–43]. Encouragingly, Rp@Al could strengthen the activation of DCs and T_H_1/T_H_2 immune responses, resulting in a strong pathogen-specific immunological memory against recurrent infections after initial treatment. This may be ascribed to the Al adjuvant, which has been reported to preferentially prime CD4^+^ T cells and further activate B cells, thereby initiating an adaptive immune response [50, 51]. The protective antimicrobial immunity was further confirmed in a *MRSA*-induced recurrent infection model. Upon second infection, Rp@Al-treated mice showed lower bacterial activity, faster abscess recovery, and a higher survival rate of up to 70% even under near-lethal doses than control mice. Furthermore, synthetic evaluation suggested that Rp@Al had adequate biosafety. Taken together, these results suggested that the adjuvant-like Rp@Al can effectively prevented recurrent infections via protective immunomodulatory effect.

These unique features of the multiplex system developed herein indicate its potential application toward the design of efficient treatment and prevention regimes for drug-resistant bacterial infections. Furthermore, it is also challenging to integrate these properties into a single system, demonstrating this could potentially stimulate further research in developing multiplex system for other applications.

## Conclusions

**Fig. 7:**
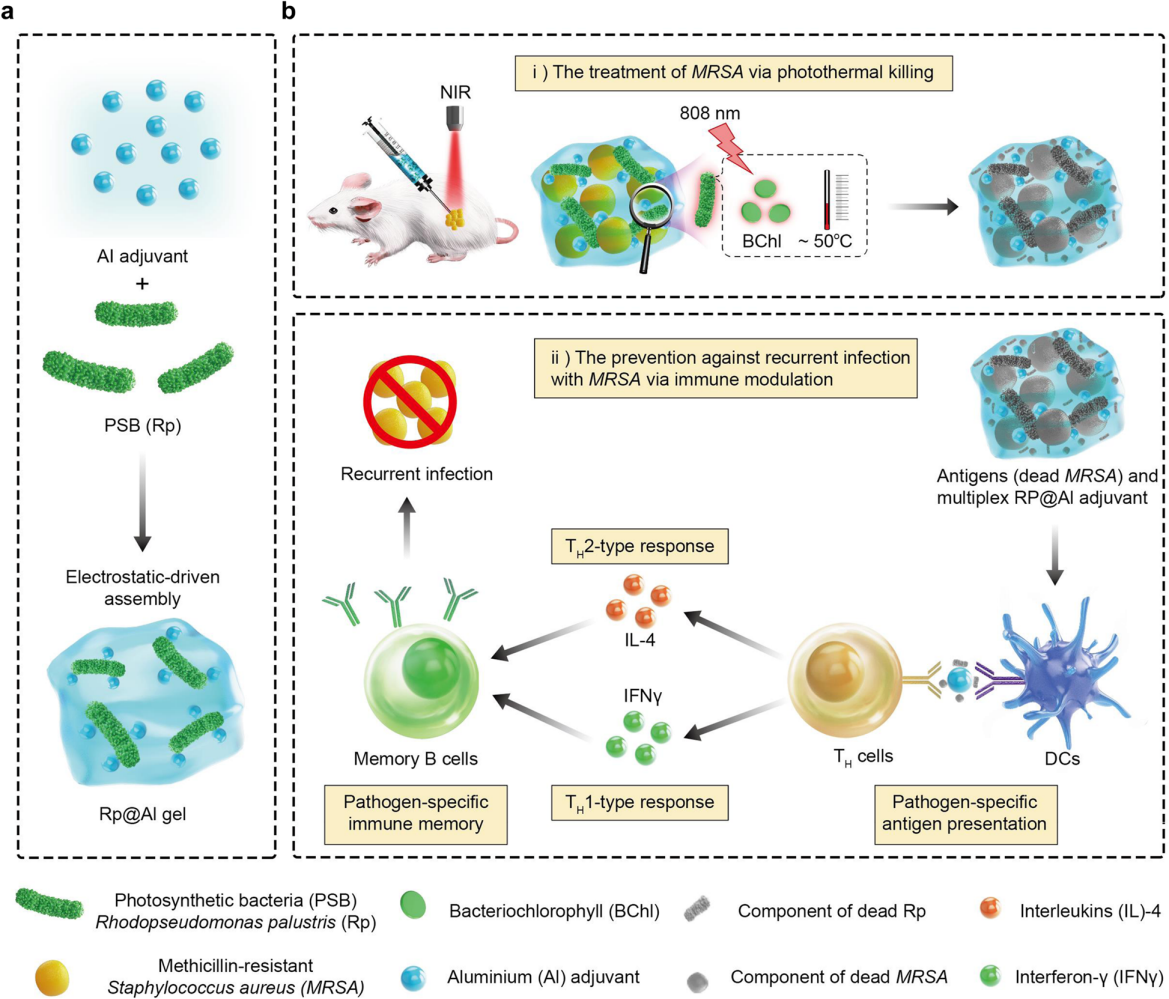
Schematic illustration of PSB-based multiplex system for the treatment of drug-resistant bacterial infections. Photosynthetic bacteria (PSB)-based Rp@Al, fabricated through an electrostatic-driven self-assembly process, containing bacteriochlorophyll (BChl) that absorbs near-infrared (NIR) light at around 805 and 865 nm, exhibited effective photothermal ablation of *MRSA*. Rp@Al also exerted an adjuvant-like effect to enhance the antigen presentation of activated DCs, thereby priming the T helper 1 (T_H_1)/T_H_2 immune response, resulting in a protective pathogen-specific immunological memory against recurrent infections with the same drug-resistant bacteria

In summary, we presented a multiplex system that utilizes photothermal ablation to achieve drug-resistant bacteria abrogation, while also preventing recurrent infections with drug-resistant bacteria through adjuvant-like modulation of the host immune response. Specifically, we i) developed a PSB-based multiplex system (Rp@Al) that was fabricated through electrostatic-driven self-assembly for combating drug-resistant bacterial infections; ii) demonstrated that Rp@Al, containing BChl, which absorbs NIR light at around 805 and 865 nm, exhibited prominent photothermal properties and photothermal ablation ability toward drug-resistant bacteria; iii) revealed that Rp@Al exerts an adjuvant-like effect to enhance the antigen presentation of activated DCs, thereby priming T_H_1/T_H_2 immune responses, resulting in a protective pathogen-specific immunological memory against recurrent infections with drug-resistant bacteria; and iv) illustrated that Rp@Al not only eliminates drug-resistant bacteria through photothermal killing but also plays a preventive role in reducing infection burden and prolonging the survival period of mice during recurrent infections (Fig. 7).

## Supplementary Information

**Additional file 1: Fig. S1.** Stability of Rp@Al on days 0, 3, and 7. **Fig. S2.** The preparation mechanism of Rp@Al. **Fig. S3.** Statistical analysis of scanning electron microscopy (SEM) images of Rp showing its diameter distribution. **Fig. S4.** Representative image of a growing Rp culture. **Fig. S5.** Time-lapse images showing the photothermal conversion of PBS, Rp, and Rp@Al. **Fig. S6.** Time-lapse images showing the photothermal conversion of PBS and different concentrations of Rp@Al. **Fig. S7.** Time-lapse images showing the photothermal conversion of Rp@Al using an 808-nm laser. **Fig. S8.** Levels of reactive oxygen species in methicillin-resistant* Staphylococcus aureus *(*MRSA*) after different treatment. **Fig. S9.** Temperature monitoring of the abscess site in mice under 808 nm laser irradiation. **Fig. S10.** Quantitative analysis showing collagen deposition levels in different treatment groups. **Fig. S11.** Representative flow scatter plot showing the percentage of CD4^+^ T cells in total leukocytes. **Fig. S12.** Comparative analysis of the proportion of memory B cells in mice from different treatment groups. **Fig. S13.** Time-lapse images showing the abscess recovery in treatment groups after second subcutaneous injection of *MRSA*. **Fig. S14.** The abscess recovery in different treatment groups after a second subcutaneous infection of *MRSA*. **Fig. S15.** Weight of the mice before intravenous re-infection with* MRSA*. **Table S1.** Summary of the photothermal conversion efficiency (η) of some photothermal agents that have been reported. **Table S2.** Summary of the pros and cons for different nanomaterials for antibacterial applications.

## Data Availability

The data that support the findings of this study are available from the corresponding authors upon reasonable request.

## Abbreviations

AMR: Antimicrobial resistance
*MRSA*: Methicillin-resistant *Staphylococcus aureus*
PSB: Photosynthetic bacteria
BChl: Bacteriochlorophyll
NIR: Near-infrared
PRRs: Pattern recognition receptors
TLRs: Toll-like receptors
NLRs: NOD-like receptors
BHI: Brain Heart Infusion
IFNγ: Interferon γ
IL: Interleukin
ELISA: Enzyme-linked immunosorbent assay
ALT: Alanine aminotransferase
AST: Aspartate aminotransferase
CRE: Creatinine
BUN: Blood urea nitrogen
TC: Total cholesterol
TG: Triglyceride
GLU: Glucose
Rp: *Rhodopseudomonas palustris*
PBS: Phosphate-buffered saline
PFA: Paraformaldehyde
DCs: Dendritic cells
H&E: Hematoxylin and eosin

## References

[1] Baym M, Stone LK, Kishony R (2016). Multidrug evolutionary strategies to reverse antibiotic resistance. Science..

[2] Theuretzbacher U, Outterson K, Engel A, Karlén A (2020). The global preclinical antibacterial pipeline. Nat Rev Microbiol.

[3] Lewis K (2020). The science of antibiotic discovery. Cell.

[4] Davies J, Davies D (2010). Origins and evolution of antibiotic resistance. Microbiol Mol Biol Rev.

[5] Murray CJ, Ikuta KS, Sharara F, Swetschinski L, Robles Aguilar G, Gray A (2022). Global burden of bacterial antimicrobial resistance in 2019: a systematic analysis. Lancet.

[6] O’Neill J. Tackling drug-resistant infections globally: final report and recommendations, Rev Antimicrob Resist. 2016. https://amr-review.org/sites/default/files/160525_Final%20paper_with%20cover.pdf.

[7] Schrader SM, Vaubourgeix J, Nathan C (2020). Biology of antimicrobial resistance and approaches to combat it. Sci Transl Med..

[8] Wu Y, Jiang W, Huo S, Li S, Xu Y, Ding S (2021). Nano-metal–organic-frameworks for treating H_2_O_2_-secreting bacteria alleviate pulmonary injury and prevent systemic sepsis. Biomaterials..

[9] Turner NA, Sharma-Kuinkel BK, Maskarinec SA, Eichenberger EM, Shah PP, Carugati M (2019). Methicillin-resistant *Staphylococcus aureus*: an overview of basic and clinical research. Nat Rev Microbiol.

[10] Steidler L, Hans W, Schotte L, Neirynck S, Obermeier F, Falk W (2000). Treatment of murine colitis by *Lactococcus lactis* secreting interleukin-10. Science.

[11] Tan L, Fu J, Feng F, Liu X, Cui Z, Li B (2020). Engineered probiotics biofilm enhances osseointegration via immunoregulation and anti-infection. Sci Adv..

[12] Yi X, Zhou H, Chao Y, Xiong S, Zhong J, Chai Z (2020). Bacteria-triggered tumor-specific thrombosis to enable potent photothermal immunotherapy of cancer. Sci Adv..

[13] Chen Q-W, Liu X, Fan J-X, Peng S, Wang J, Wang X (2020). Self-mineralized photothermal bacteria hybridizing with mitochondria-targeted metal–organic frameworks for augmenting photothermal tumor therapy. Adv Funct Mater.

[14] Zhong D, Li W, Qi Y, He J, Zhou M (2020). Photosynthetic biohybrid nanoswimmers system to alleviate tumor hypoxia for FL/PA/MR imaging-guided enhanced radio-photodynamic synergetic therapy. Adv Funct Mater.

[15] Li W, Wang S, Zhong D, Du Z, Zhou M (2021). A bioactive living hydrogel: photosynthetic bacteria mediated hypoxia elimination and bacteria-killing to promote infected Wound healing. Adv Therap.

[16] Wang S-B, Liu X-H, Li B, Fan J, Ye J, Cheng H (2019). Bacteria-assisted selective photothermal therapy for precise tumor inhibition. Adv Funct Mater.

[17] Qiao Y, Yang F, Xie T, Du Z, Zhong D, Qi Y (2020). Engineered algae: a novel oxygen-generating system for effective treatment of hypoxic cancer. Sci Adv..

[18] Zhong D, Zhang D, Xie T, Zhou M (2020). Biodegradable microalgae-based carriers for targeted delivery and imaging-guided therapy toward lung metastasis of breast cancer. Small..

[19] Riglar DT, Silver PA (2018). Engineering bacteria for diagnostic and therapeutic applications. Nat Rev Microbiol.

[20] Huang X, Pan J, Xu F, Shao B, Wang Y, Guo X (2021). Bacteria-based cancer immunotherapy. Adv Sci (Weinh).

[21] Zheng JH, Nguyen VH, Jiang SN, Park SH, Tan W, Hong SH (2017). Two-step enhanced cancer immunotherapy with engineered *Salmonella typhimurium* secreting heterologous flagellin. Sci Transl Med..

[22] Gao C, Wang Q, Li J, Kwong CHT, Wei J, Xie B (2022). In vivo hitchhiking of immune cells by intracellular self-assembly of bacteria-mimetic nanomedicine for targeted therapy of melanoma. Sci Adv..

[23] Chowdhury S, Castro S, Coker C, Hinchliffe TE, Arpaia N, Danino T (2019). Programmable bacteria induce durable tumor regression and systemic antitumor immunity. Nat Med.

[24] Zheng DW, Chen Y, Li ZH, Xu L, Li CX, Li B (2018). Optically controlled bacterial metabolite for cancer therapy. Nat Commun.

[25] Gareau MG, Sherman PM, Walker WA (2010). Probiotics and the gut microbiota in intestinal health and disease. Nat Rev Gastroenterol Hepatol.

[26] Servin AL, Liévin-Le Moal VL-L (2014). Anti-infective activities of lactobacillus strains in the human intestinal microbiota: from probiotics to gastrointestinal anti-infectious biotherapeutic agents. Clin Microbiol Rev..

[27] Liu X, Huang L, Rensing C, Ye J, Nealson KH, Zhou S (2021). Syntrophic interspecies electron transfer drives carbon fixation and growth by *Rhodopseudomonas palustris* under dark, anoxic conditions. Sci Adv..

[28] Lu H, Zhang G, Wan T, Lu Y (2011). Influences of light and oxygen conditions on photosynthetic bacteria macromolecule degradation: different metabolic pathways. Bioresour Technol.

[29] Lu H, Zhang G, Zheng Z, Meng F, Du T, He S (2019). Bio-conversion of photosynthetic bacteria from non-toxic wastewater to realize wastewater treatment and bioresource recovery: a review. Bioresour Technol.

[30] Shu X, Royant A, Lin MZ, Aguilera TA, Lev-Ram V, Steinbach PA (2009). Mammalian expression of infrared fluorescent proteins engineered from a bacterial phytochrome. Science.

[31] Ryu MH, Kang IH, Nelson MD, Jensen TM, Lyuksyutova AI, Siltberg-Liberles J (2014). Engineering adenylate cyclases regulated by near-infrared window light. Proc Natl Acad Sci U S A.

[32] Zheng P, Fan M, Liu H, Zhang Y, Dai X, Li H (2021). Self-propelled and near-infrared-phototaxic photosynthetic bacteria as photothermal agents for hypoxia-targeted cancer therapy. ACS Nano.

[33] Makabenta JMV, Nabawy A, Li CH, Schmidt-Malan S, Patel R, Rotello VM (2021). Nanomaterial-based therapeutics for antibiotic-resistant bacterial infections. Nat Rev Microbiol.

[34] Guo B, Huang Z, Shi Q, Middha E, Xu S, Li L (2020). Organic small molecule based photothermal agents with molecular rotors for malignant breast cancer therapy. Adv Funct Mater.

[35] Zhang L, Liu Y, Huang H, Xie H, Zhang B, Xia W (2022). Multifunctional nanotheranostics for near infrared optical imaging-guided treatment of brain tumors. Adv. Drug Deliv. Rev..

[36] Zhang L, Forgham H, Huang X, Shen A, Davis TP, Qiao R (2022). All-in-one inorganic nanoagents for near-infrared-II photothermal-based cancer theranostics. Materials Today Advances..

[37] Hancock REW, Nijnik A, Philpott DJ (2012). Modulating immunity as a therapy for bacterial infections. Nat Rev Microbiol.

[38] Pernet E, Guillemot L, Burgel PR, Martin C, Lambeau G, Sermet-Gaudelus I (2014). *Pseudomonas aeruginosa* eradicates *Staphylococcus aureus* by manipulating the host immunity. Nat Commun.

[39] Kim B, Pang HB, Kang J, Park JH, Ruoslahti E, Sailor MJ (2018). Immunogene therapy with fusogenic nanoparticles modulates macrophage response to *Staphylococcus aureus*. Nat Commun.

[40] Tang H, Qu X, Zhang W, Chen X, Zhang S, Xu Y (2022). Photosensitizer nanodot eliciting immunogenicity for photo-immunologic therapy of postoperative methicillin-resistant *Staphylococcus aureus* infection and secondary recurrence. Adv Mater..

[41] Fu J, Li Y, Zhang Y, Liang Y, Zheng Y, Li Z (2021). An engineered pseudo-macrophage for rapid treatment of bacteria-infected osteomyelitis via microwave-excited anti-infection and immunoregulation. Adv Mater..

[42] Wei X, Ran D, Campeau A, Xiao C, Zhou J, Dehaini D (2019). Multiantigenic nanotoxoids for antivirulence vaccination against antibiotic-resistant gram-negative bacteria. Nano Lett.

[43] Wang C, Xiao Y, Zhu W, Chu J, Xu J, Zhao H (2020). Photosensitizer-modified MnO_2_ nanoparticles to enhance photodynamic treatment of abscesses and boost immune protection for treated mice. Small..

[44] Ong PY, Ohtake T, Brandt C, Strickland I, Boguniewicz M, Ganz T (2002). Endogenous antimicrobial peptides and skin infections in atopic dermatitis. N Engl J Med.

[45] Hennessy EJ, Parker AE, O’Neill LAJ (2010). Targeting toll-like receptors: emerging therapeutics?. Nat Rev Drug Discov.

[46] Sorbara MT, Philpott DJ (2011). Peptidoglycan: a critical activator of the mammalian immune system during infection and homeostasis. Immunol Rev.

[47] Dunne A, O’Neill LAJ (2003). The interleukin-1 receptor/toll-like receptor superfamily: signal transduction during inflammation and host defense. Sci STKE..

[48] Zaheer SA, Mukherjee R, Ramkumar B, Misra RS, Sharma AK, Kar HK (1993). Combined multidrug and *Mycobacterium w* vaccine therapy in patients with multibacillary leprosy. J Infect Dis.

[49] Klinman DM, Xie H, Ivins BE (2006). CpG oligonucleotides improve the protective immune response induced by the licensed anthrax vaccine. Ann N Y Acad Sci.

[50] Marrack P, McKee AS, Munks MW (2009). Towards an understanding of the adjuvant action of aluminium. Nat Rev Immunol.

[51] Eisenbarth SC, Colegio OR, O’Connor W, Sutterwala FS, Flavell RA (2008). Crucial role for the Nalp3 inflammasome in the immunostimulatory properties of aluminium adjuvants. Nature.

[52] Jiang H, Wang Q, Li L, Zeng Q, Li H, Gong T (2018). Turning the old adjuvant from gel to nanoparticles to amplify CD8^+^ T cell responses. Adv Sci (Weinh).

[53] Hessel CM, Pattani VP, Rasch M, Panthani MG, Koo B, Tunnell JW (2011). Copper selenide nanocrystals for photothermal therapy. Nano Lett.

[54] Li Y, Bai G, Zeng S, Hao J (2019). Theranostic carbon dots with innovative NIR-II emission for in vivo renal-excreted optical imaging and photothermal therapy. ACS Appl Mater Interfaces.

[55] Sun Z, Xie H, Tang S, Yu XF, Guo Z, Shao J (2015). Ultrasmall black phosphorus quantum dots: synthesis and use as photothermal agents. Angew Chem Int Ed Engl.

[56] Ding Y, Wang C, Lu B, Yao Y (2021). Enhancing the stability and photothermal conversion efficiency of ICG by pillar[5]arene-Based Host-Guest Interaction. Front Chem..

[57] Bellier N, Baipaywad P, Ryu N, Lee JY, Park H (2022). Recent biomedical advancements in graphene oxide- and reduced graphene oxide-based nanocomposite nanocarriers. Biomater Res.

[58] Huang H, Ali A, Liu Y, Xie H, Ullah S, Roy S (2023). Advances in image-guided drug delivery for antibacterial therapy. Adv. Drug Deliv. Rev..

[59] Quintero-Quiroz C, Acevedo N, Zapata-Giraldo J, Botero LE, Quintero J, Zárate-Triviño D (2019). Optimization of silver nanoparticle synthesis by chemical reduction and evaluation of its antimicrobial and toxic activity. Biomater Res.

[60] Abebe F (2019). Synergy between Th1 and Th2 responses during *Mycobacterium tuberculosis* infection: a review of current understanding. Int Rev Immunol.

[61] Mullard A (2022). The deadly burden of drug-resistant bacteria. Nat Rev Drug Discov.

[62] Zhu Y, Hao W, Wang X, Ouyang J, Deng X, Yu H (2022). Antimicrobial peptides, conventional antibiotics, and their synergistic utility for the treatment of drug-resistant infections. Med Res Rev.

