# Supplementary material for: Bacteria-based multiplex system eradicates recurrent infections with drug-resistant bacteria via photothermal killing and protective immunity elicitation
Source: Biomater Res. 2023 Apr 6;27:27. doi: 10.1186/s40824-023-00363-0 (PMC10080897; doi:10.1186/s40824-023-00363-0)
Supplement: Supplementary file 1 — Additional file 1: Fig. S1. Stability of Rp@Al on days 0, 3, and 7. Fig. S2. The preparation mechanism of Rp@Al. Fig. S3. Statistical analysis of scanning electron microscopy (SEM) images of Rp showing its diameter distribution. Fig. S4. Representative image of a growing Rp culture. Fig. S5. Time-lapse images showing the photothermal conversion of PBS, Rp, and Rp@Al. Fig. S6. Time-lapse images showing the photothermal conversion of PBS and different concentrations of Rp@Al. Fig. S7. Time-lapse images showing the photothermal conversion of Rp@Al using an 808-nm laser. Fig. S8. Levels of reactive oxygen species in methicillin-resistant Staphylococcus aureus (MRSA) after different treatment. Fig. S9. Temperature monitoring of the abscess site in mice under 808 nm laser irradiation. Fig. S10. Quantitative analysis showing collagen deposition levels in different treatment groups. Fig. S11. Representative flow scatter plot showing the percentage of CD4+ T cells in total leukocytes. Fig. S12. Comparative analysis of the proportion of memory B cells in mice from different treatment groups. Fig. S13. Time-lapse images showing the abscess recovery in treatment groups after second subcutaneous injection of MRSA. Fig. S14. The abscess recovery in different treatment groups after a second subcutaneous infection of MRSA. Fig. S15. Weight of the mice before intravenous re-infection with MRSA. Table S1. Summary of the photothermal conversion efficiency (η) of some photothermal agents that have been reported. Table S2. Summary of the pros and cons for different nanomaterials for antibacterial applications. [file 40824_2023_363_MOESM1_ESM.docx]

**Additional File**

**Supplementary Information**

**Bacteria-based multiplex system eradicates recurrent infections with drug-resistant bacteria via photothermal killing and protective immunity elicitation**

Youcui Xu, Yi Wu, Yi Hu, Mengran Xu, Yanyan Liu, Yuting Ding, Jing Chen, Xiaowan Huang, Longping Wen, Jiabin Li, Chen Zhu

**Supplementary figures**


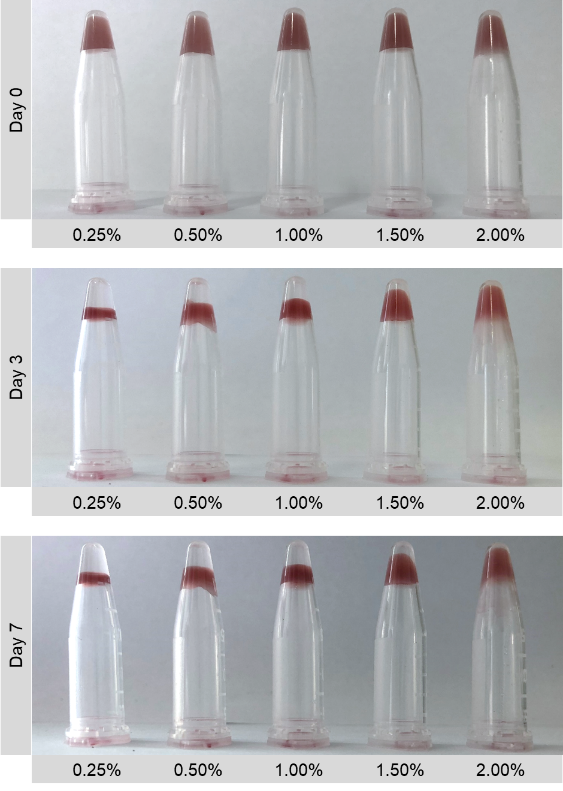


Fig. S1. Stability of Rp@Al on days 0, 3, and 7. Rp@Al was prepared using different concentrations of aluminum (Al) adjuvant and *Rhodopseudomonas palustris* (Rp).


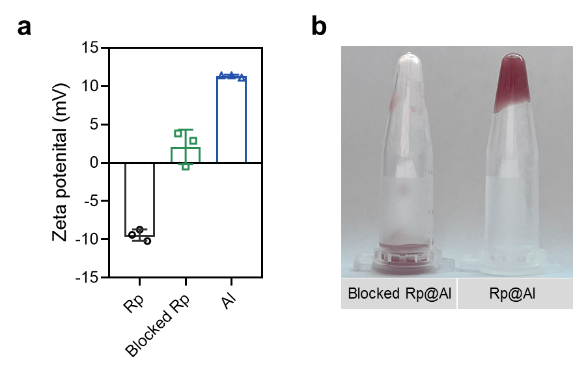


Fig. S2. The preparation mechanism of Rp@Al. Zeta potential (a) and images (b) showing the preparation mechanism of Rp@Al. Mean ± SD, n = 3.


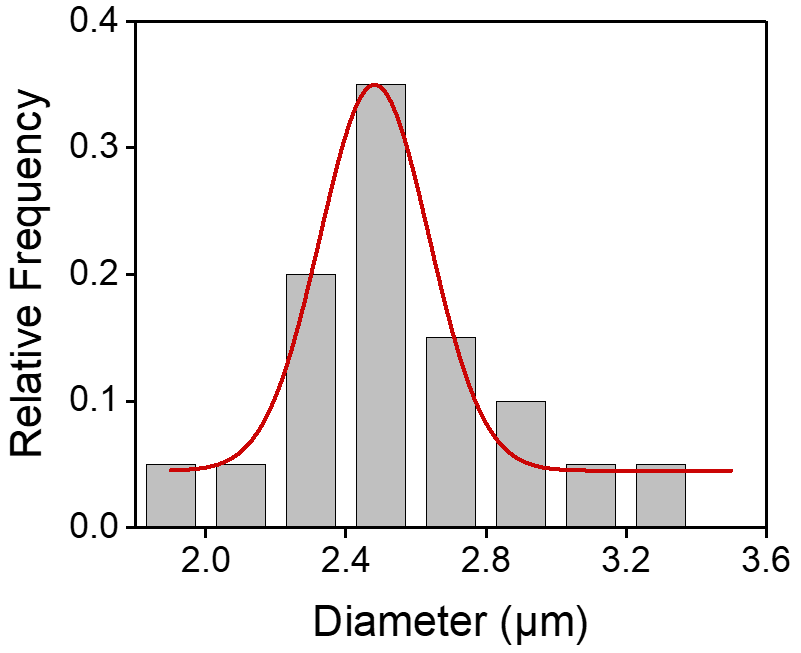


Fig. S3. Statistical analysis of scanning electron microscopy (SEM) images of Rp showing its diameter distribution. n = 20.


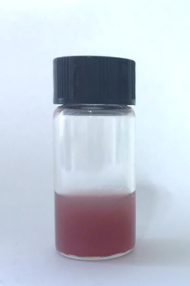


Fig. S4. Representative image of a growing Rp culture.


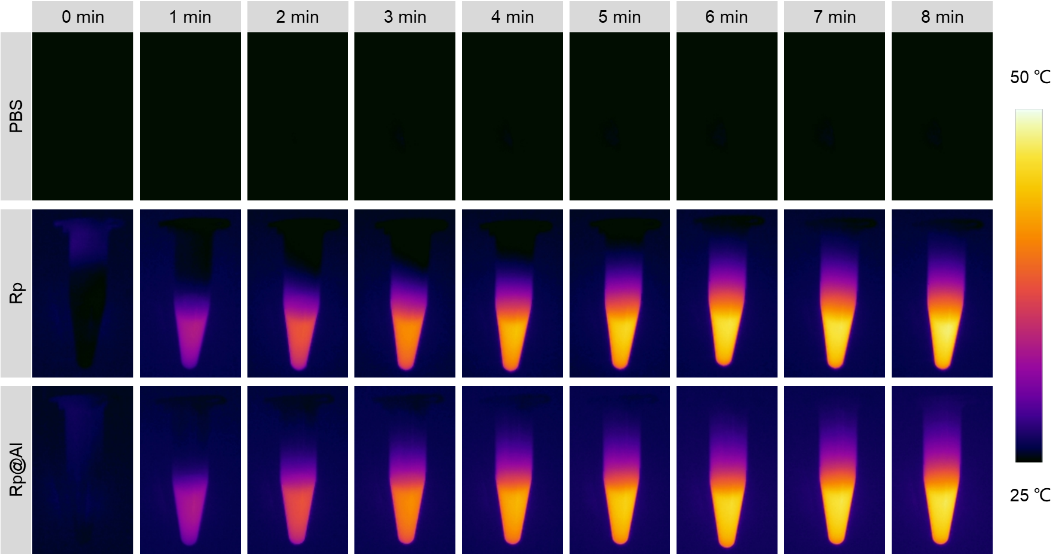


Fig. S5. Time-lapse images showing the photothermal conversion of PBS, Rp, and Rp@Al. These images were obtained using an 808-nm laser with a power density of 2 W cm^-2^ for 8 min. PBS, phosphate-buffered saline.


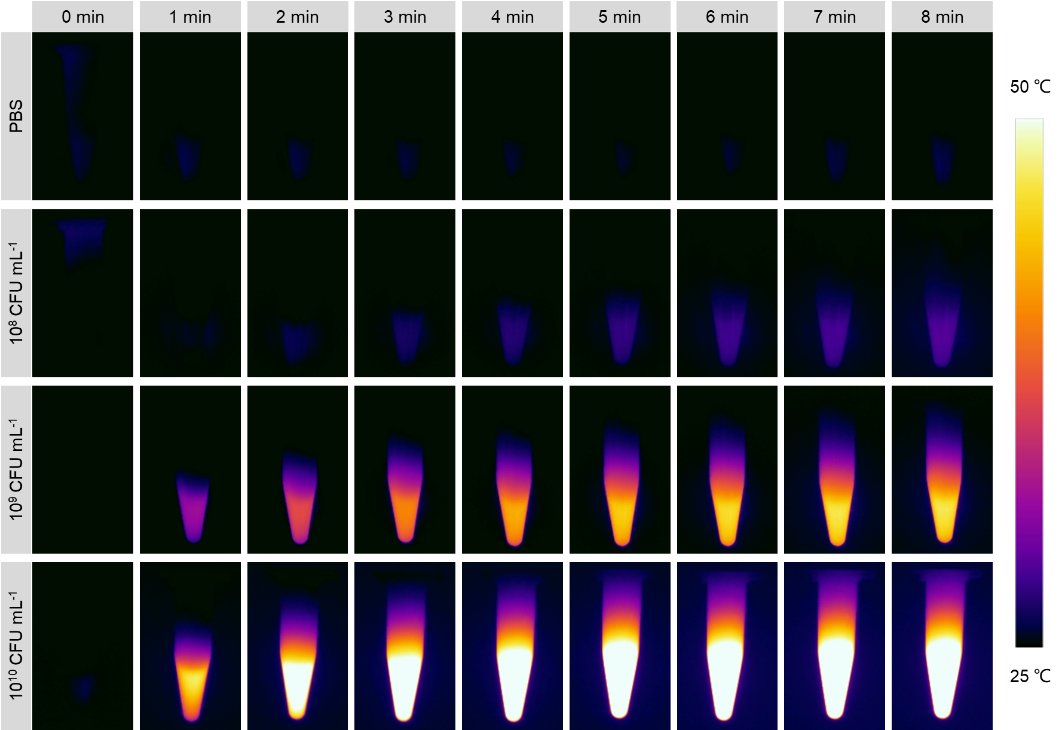


Fig. S6. Time-lapse images showing the photothermal conversion of PBS and different concentrations of Rp@Al. These images were obtained using an 808-nm laser with a power density of 2 W cm^-2^ for 8 min.


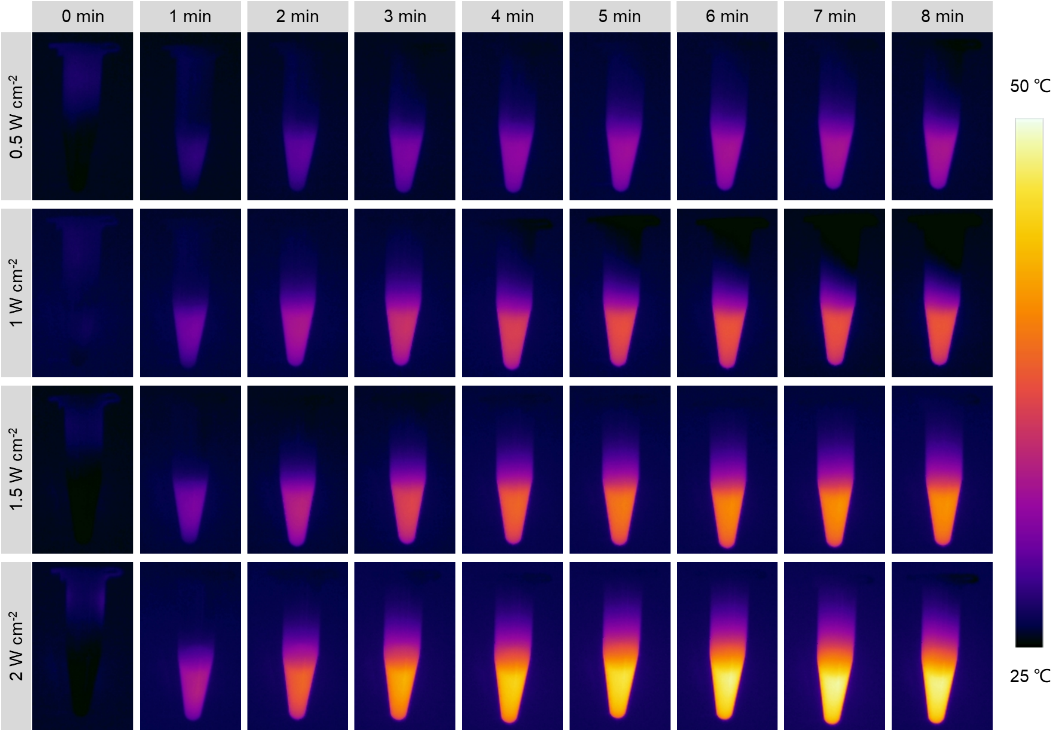


Fig. S7. Time-lapse images showing the photothermal conversion of Rp@Al using an 808-nm laser. These images were obtained with different power densities for 8 min.


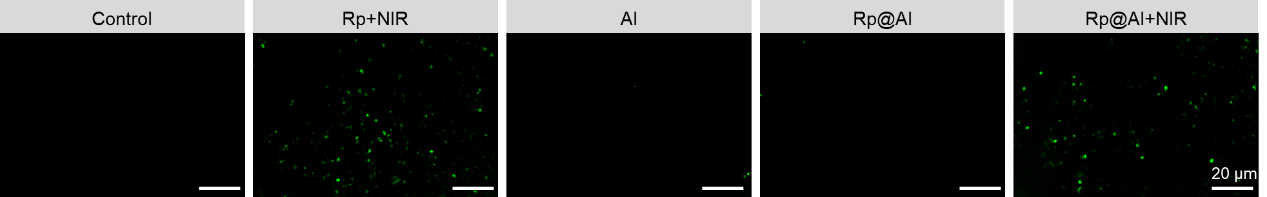


Fig. S8. Levels of reactive oxygen species in methicillin-resistant *Staphylococcus aureus* (*MRSA*) after different treatment.


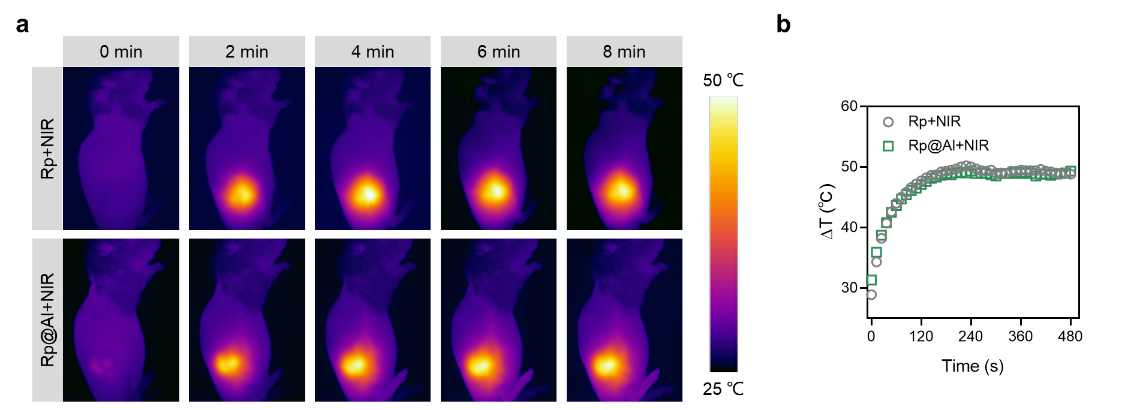


Fig. S9. Temperature monitoring of the abscess site in mice under 808 nm laser irradiation. Time-lapse images (a) and photothermal temperature curves (b) showing the photothermal conversion at the abscess site in mice under an 808 nm laser with a power density of 1.5 W cm^-2^ for 8 min.

Fig. S10. Quantitative analysis showing collagen deposition levels in different treatment groups. Mean ± SEM, n = 5. ns, no significance, ****P* < 0.001, and **** *P* < 0.0001.


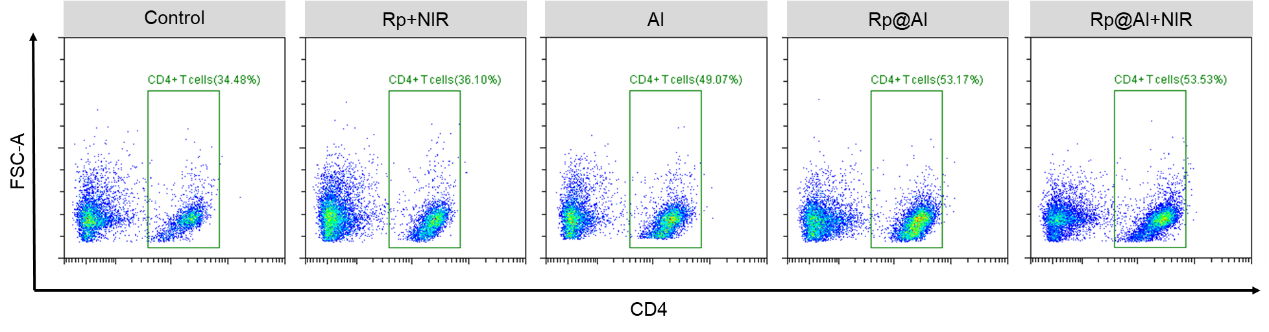


Fig. S11. Representative flow scatter plot showing the percentage of CD4^+^ T cells in total leukocytes. The leukocytes isolated from lymph nodes were harvested from mice in different treatment groups.


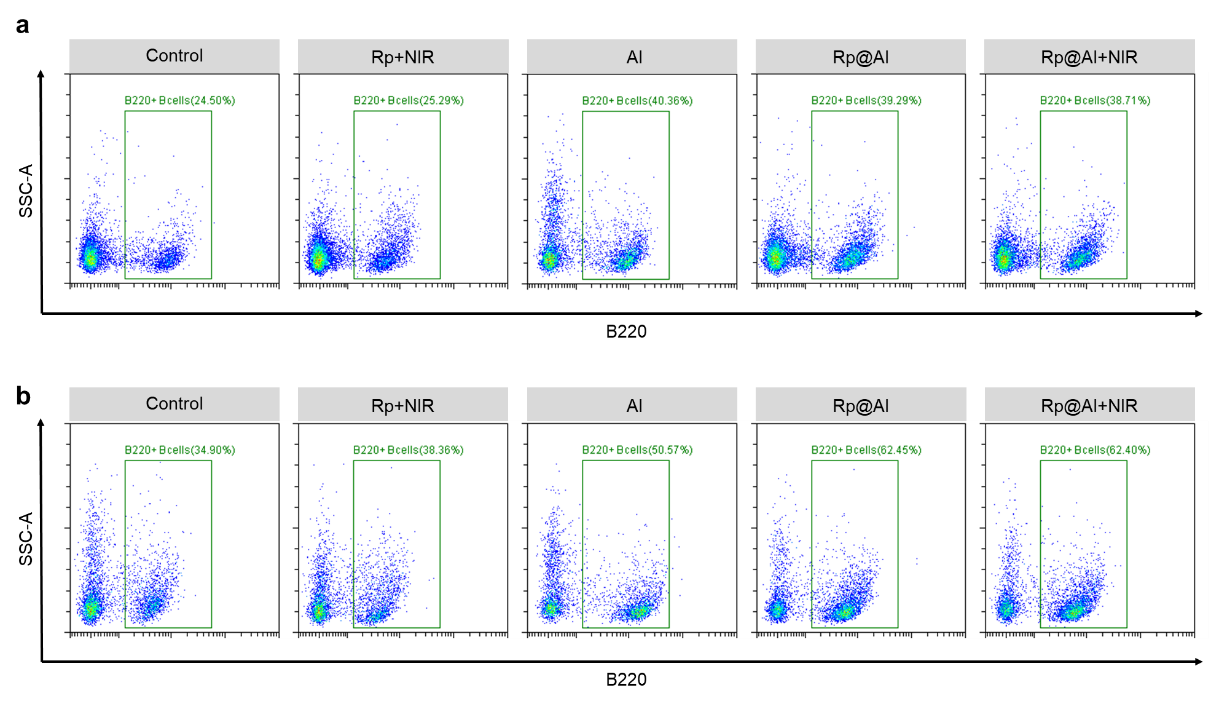


Fig. S12. Comparative analysis of the proportion of memory B cells in mice from different treatment groups. Representative flow scatter plot showing the percentage of B220^+^ B cells in total leukocytes isolated from lymph nodes (a) and spleens (b) harvested from different treatment groups.


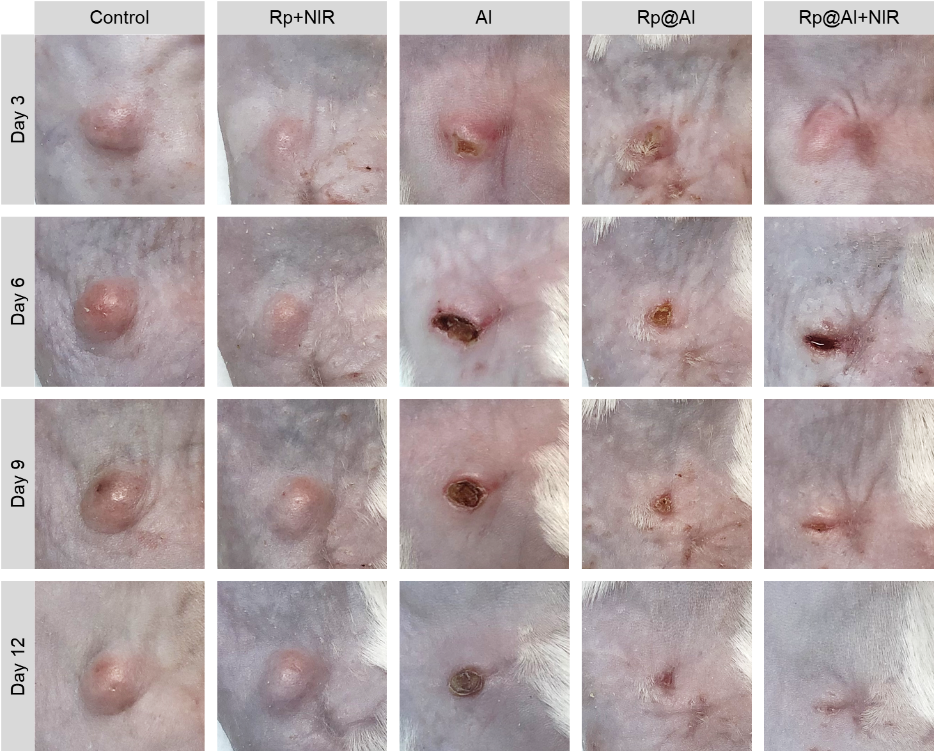


Fig. S13. Time-lapse images showing the abscess recovery in treatment groups after second subcutaneous injection of *MRSA*.


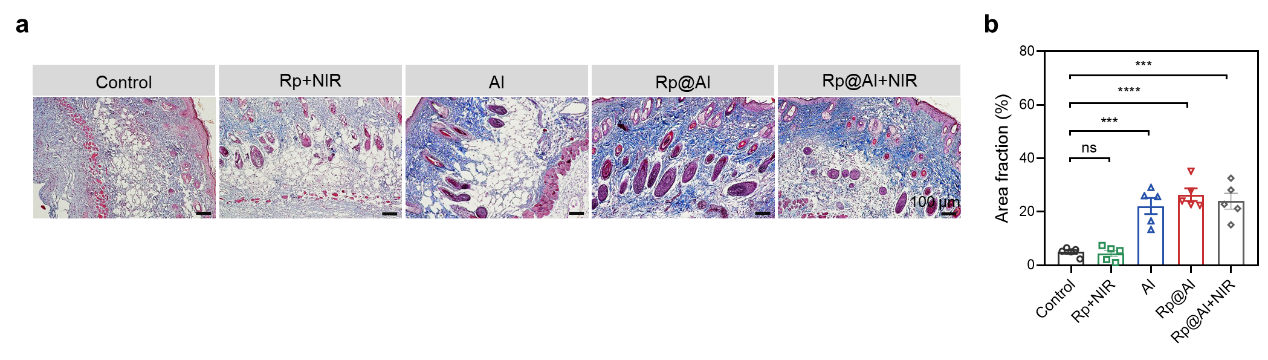


Fig. S14. The abscess recovery in different treatment groups after a second subcutaneous infection of *MRSA*. Representative images of Masson’s trichrome staining (a) and quantitative analysis (b) showing collagen deposition levels in different treatment groups. Mean ± SEM, n = 5. ns, no significance, ****P* < 0.001, and *****P* < 0.0001.

Fig. S15. Weight of the mice before intravenous re-infection with *MRSA*. Mean ± SEM, n = 10. ns, no significance.


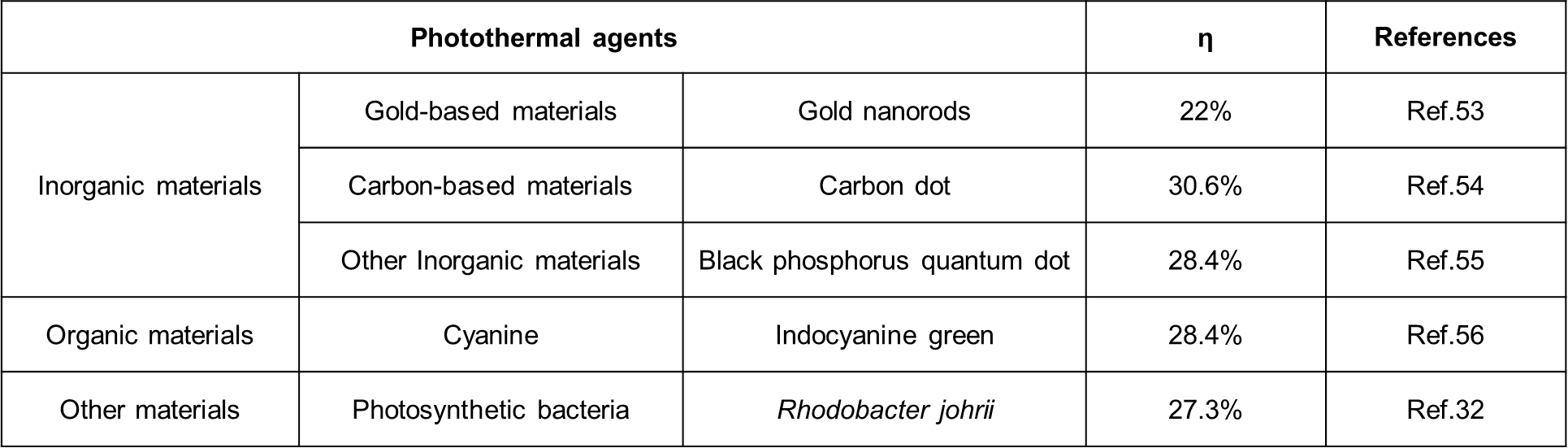


Table S1. Summary of the photothermal conversion efficiency (η) of some photothermal agents that have been reported.


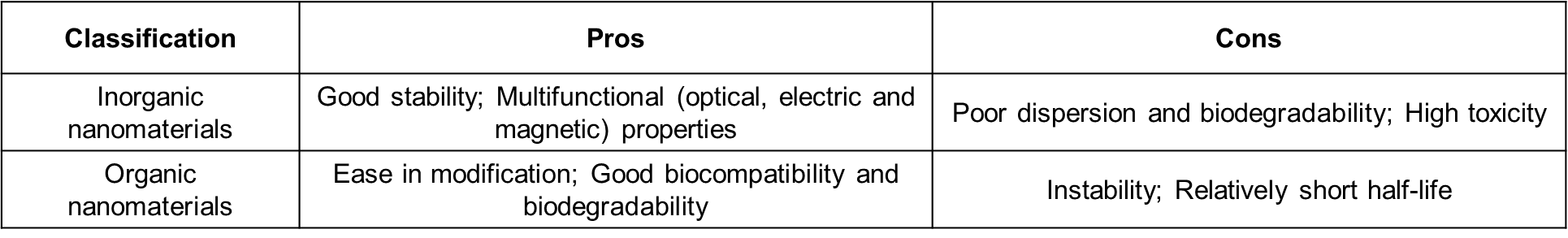


**Table S2. Summary of the pros and cons for different nanomaterials for antibacterial applications.**
